# Proteins from toad’s parotoid macroglands: do they play a role in gland functioning and chemical defence?

**DOI:** 10.1186/s12983-023-00499-8

**Published:** 2023-06-16

**Authors:** Krzysztof Kowalski, Paweł Marciniak, Leszek Rychlik

**Affiliations:** 1 Department of Vertebrate Zoology and Ecology, Faculty of Biological and Veterinary Sciences, Institute of Biology, Nicolaus Copernicus University, Lwowska 1, 87-100 Toruń, Poland; 2 Department of Animal Physiology and Developmental Biology, Faculty of Biology, Institute of Experimental Biology, Adam Mickiewicz University, Uniwersytetu Poznańskiego 6, 61-614 Poznań, Poland; 3 Department of Systematic Zoology, Faculty of Biology, Institute of Environmental Biology, Adam Mickiewicz University, Uniwersytetu Poznańskiego 6, 61-614 Poznań, Poland

**Keywords:** Anurans, *Bufo bufo*, Bufonids, Cholesterol synthesis, IDI1, Phosphomevalonate kinase, Toad poison

## Abstract

**Background:**

Parotoid gland secretion of bufonid toads is a rich source of toxic molecules that are used against predators, parasites and pathogens. Bufadienolides and biogenic amines are the principal compounds responsible for toxicity of parotoid secretion. Many toxicological and pharmacological analyses of parotoid secretions have been performed, but little is known about the processes related to poison production and secretion. Therefore, our aim was to investigate protein content in parotoids of the common toad, *Bufo bufo*, to understand the processes that regulate synthesis and excretion of toxins as well as functioning of parotoid macroglands.

**Results:**

Applying a proteomic approach we identified 162 proteins in the extract from toad’s parotoids that were classified into 11 categories of biological functions. One-third (34.6%) of the identified molecules, including acyl-CoA-binding protein, actin, catalase, calmodulin, and enolases, were involved in cell metabolism. We found many proteins related to cell division and cell cycle regulation (12.0%; e.g. histone and tubulin), cell structure maintenance (8.4%; e.g. thymosin beta-4, tubulin), intra- and extracellular transport (8.4%), cell aging and apoptosis (7.3%; e.g. catalase and pyruvate kinase) as well as immune (7.0%; e.g. interleukin-24 and UV excision repair protein) and stress (6.3%; including heat shock proteins, peroxiredoxin-6 and superoxide dismutase) response. We also identified two proteins, phosphomevalonate kinase and isopentenyl-diphosphate delta-isomerase 1, that are involved in synthesis of cholesterol which is a precursor for bufadienolides biosynthesis. STRING protein-protein interaction network predicted for identified proteins showed that most proteins are related to metabolic processes, particularly glycolysis, stress response and DNA repair and replication. The results of GO enrichment and KEGG analyses are also consistent with these findings.

**Conclusion:**

This finding indicates that cholesterol may be synthesized in parotoids, and not only in the liver from which is then transferred through the bloodstream to the parotoid macroglands. Presence of proteins that regulate cell cycle, cell division, aging and apoptosis may indicate a high epithelial cell turnover in parotoids. Proteins protecting skin cells from DNA damage may help to minimize the harmful effects of UV radiation. Thus, our work extends our knowledge with new and important functions of parotoids, major glands involved in the bufonid chemical defence.

**Supplementary Information:**

The online version contains supplementary material available at 10.1186/s12983-023-00499-8.

## Background

**Fig. 1:**
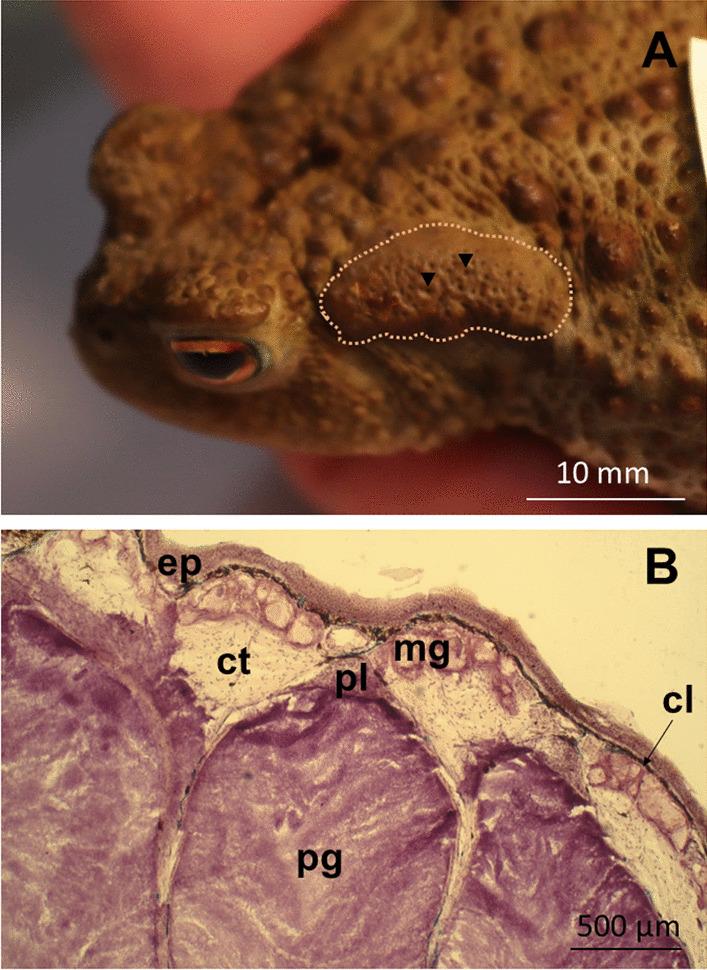
**A** Female common toad *Bufo bufo* exhibiting the left parotoid macrogland (dashed line) localized at the postorbital region. Black triangles point to the glandular pores. **B** Longitudinal section of parotoid bottle-shaped glands. Abbreviations: cl—chromatophore layer; ct—connective tissue; ep—epidermis; mg—mucous glands; pg—poison glands; pl—epithelial plug. Photos by Anna Kowalczewska (**A**) and Krzysztof Kowalski (**B**)

Amphibians inhabit a diverse range of environments worldwide [1, 2]. As amphibiotic organisms they require water reservoirs to complete their larval development. After metamorphosis most species, like bufonid toads, are usually terrestrial [1]. The migration from an aquatic to a more hybrid (semiaquatic and terrestrial) environment led to many unique traits in amphibian evolution. For instance, amphibian skin plays a crucial role in their adaptation to the terrestrial environment, being responsible for respiration, water balance regulation, excretion, temperature control, osmotic changes, reproduction, and also defence [3–5]. The tegument contains two types of specialized glands, termed mucous and granular (or poison) glands. The latter form in bufonids large parotoid macroglands located in the postorbital region (Fig. 1A) [3, 4] and are involved in chemical defence [6–8].

Poison secreted from parotoids is used to protect against fungi, microorganisms and predators [9–14], as it may be irritating and even lethal for the latter [6, 13]. The chemical compounds contained in parotoid secretions have been classified into four main categories: (i) biogenic amines, such as adrenaline, noradrenaline, bradykinin, or histamine; (ii) bufadienolides; (iii) alkaloids, such as batrachotoxin or tetrodotoxin, and (iv) peptides and proteins [4, 5, 11–13, 15]. Bufadienolides are the major components of the bufonid parotoid secretion responsible for its toxicity [8, 11, 15]. According to Steyn and Heerden [16], the toad gland secretion may contain up to 86 types of bufadienolides, including arenobufagin, bufalin, bufogenin, bufotalin, cinobufagin, cinobufotalin, gamabufotalin, marinobufagin, and telocinobufagin [8, 11, 17].

Recently, some peptides and proteins have been identified in the toad parotoid secretions [12, 13, 18–24], but their abundance seems to be much lower than those of bufadienolides and biogenic amines [4, 12]. For instance, Sousa-Filho et al. [20] obtained 104 proteins, including actin, beta-actin, ribosomal proteins, catalase, galectin, and uncharacterised proteins, in the *Rhinella schneideri* parotoid secretion by proteomic analysis. No peptides were found. Huo et al. [25] identified 939 unique peptides by de novo approach in parotoid macrogland secretion of *Bufo gargarizans*. Mariano et al. [23] identified 42 proteins and sequenced de novo 153 peptides in the *Duttaphrynus melanostictus* skin secretion. The most common proteins were acyl-CoA-binding protein, alcohol dehydrogenase, calmodulin, catalase, galectin, proteasome subunit alpha- and beta-type, and histone. It is assumed that bufonid parotoid gland secretions do not contain bioactive peptides similar to those from the skin of many frog species [4, 12]. It has been also suggested that peptides from parotoid secretion of *Rhinella marina* could be breakdown products of larger proteins that ensure the proper gland functioning [12, 23]. However, our previous results indicate that some proteins from parotoid secretion of *Bufo bufo* may participate in the toad chemical defence [13].

Most studies of bufonid chemical defence focused on the biochemistry and pharmacology of parotoid gland secretions. Little is known about the functioning of parotoid macroglands and processes that regulate toxin biosynthesis, poison production and excretion. Therefore, to understand these processes, in this work we aimed to investigate protein content in the extract from parotoid macroglands. As a model species we used the common toad, *Bufo bufo*, which is known for producing potent poison in its parotoids [13, 26]. Some proteins and peptides, such as muscle creatine kinase, proteasome subunit α type, phospholipid hydroperoxide glutathione peroxidase, cytotoxic T-lymphocyte protein, serine/threonine-protein kinase, so far have successfully been identified in this species [13].

## Materials and methods

### Toads trapping and housing

Trapping of toads was performed from March to April in 2016. Adult female toads *Bufo bufo* were captured (using 10-L pitfalls and drift fences) in gardens and parks in Poznań (western Poland), placed into transporters and carried to the laboratory. Then, they were placed into large (46 × 30 × 28 cm, 39 l) aqua-terraria equipped with bedding (a mixture of peat and sand). The terraria were regularly irrigated to maintain the adequate humidity. Each terrarium contained a shelter (flowerpot) and a water tank to allow the toads to submerge in water. Food (mealworms and crickets) and water were provided ad libitum. The toads were kept in the breeding room under standard laboratory conditions (temperature: 21 °C; humidity: 65–70%; artificial photoperiod: 12L:12D).

### Extraction of toad parotoid macroglands and sample preparation

Two toads were decapitated and their parotoid glands were carefully isolated (to not squeeze them and not release the poison) and transferred into 600 µl of methanol. Tissues were next homogenized and samples were centrifuged at 10,000 × g and 4 °C for 30 min. The supernatants were collected, and the protein content was determined using a Direct Detect spectrometer (MERCK Millipore, Warsaw, Poland). Then, the supernatants were used for peptide analysis by reverse phase high-performance liquid chromatography (RP-HPLC). Separation was performed using a Dionex Ultimate 3000 chromatographic system comprising a dual pump programmable solvent module. Supernatants were analysed using a BioBasic-18 analytical column (5 µm, 150 × 4.6 mm; Thermo Scientific). The samples were eluted with a gradient of 5–60% acetonitrile (ACN)/0.1% TFA with a flow rate 0.5 ml/min for 55 min. The eluent was monitored at 214 nm and fractions were collected into 1.5-ml tubes.

### Protein identification and analysis of their biological functions

Peptides and proteins from the methanolic extract of the toad parotoid glands were analysed by liquid chromatography coupled to tandem mass spectrometry (LC-MS/MS) using a Nano-Acquity LC system (Waters, Milford, Massachusetts, USA) and an OrbitrapVelos mass spectrometer (Thermo Electron Corp., San Jose, CA). Before performing the analysis, the proteins were subjected to an ion-solution digestion procedure. Proteins were: (1) reduced with 50 mM TCEP for 30 min at 60 °C, (2) alkylated with 200 mM MMTA for 30 min at room temperature and (3) digested overnight with trypsin (sequencing Grade Modified Trypsin—Promega V5111). Next, the samples were applied to an RP-18 precolumn (nanoACQUITY Symmetry® C18—Waters 186,003,514) using water containing 0.1% TFA as a mobile phase and were transferred to a nano-HPLC RP-18 column (nanoACQUITY BEH C18—Waters 186,003,545). The samples were eluted with a gradient of 0–35% acetonitrile in the presence of 0.05% formic acid with a flow rate of 250 nl/min for 180 min. The column was directly coupled to the ion source of the spectrometer working within data dependent on the MS to MS/MS switch. To ensure a lack of cross contamination from previous samples, each analysis was preceded by a blank run.

The proteins were identified by a Mascot Search (Matrix Science, London, UK) against the SwissProt database. The search parameters were as follows: type of search: MS/MS Ion Search; enzyme specificity: trypsin; fixed methylthio modification of cysteine; variable modifications: methionine oxidation; mass values: monoisotopic; protein mass: unrestricted; peptide mass tolerance: 20 ppm; fragment mass tolerance: 0.1 D; number of missed cleavage sites allowed: 1; instrument type: HCD. Peptides with Mascot scores exceeding the threshold value of *p* < 0.05 were considered positively identified. The protein content was calculated based on the Exponentially Modified Protein Abundance Index (emPAI) [27].

The biological functions of the identified proteins were determined by searching the UniProt database. All functions were then classified into 11 categories (Table 1). The percentage of proteins displaying particular functions from each category was calculated. To understand the cellular processes in toad’s parotoids, protein-protein interaction networks were built using STRING database [28]. Because molecular data on bufonid toads are scarce, predicted interactions between identified in this work proteins were analysed based on homology to the western clawed frog *Xenopus tropicalis*, a common anuran model. Then, Gene ontology (GO) enrichment analysis was performed to predict which metabolic processes the identified proteins are involved in, and what are their molecular functions (GO molecular function analysis). KEGG analysis was carried out to predict which signalling pathways the identified proteins participate in. Finally, GO component analysis was performed to predict the localisation of particular proteins within the cell.

**Table 1:** Categories of biological functions and number of proteins identified in the extract from parotoids of the common toad *Bufo bufo* displaying particular functions

Function category	Biological function	No. of proteins
1—Cell division & cell cycle regulation	Cell division, cell cycle cytokinesis, cell proliferation, cell cycle regulation/control/progression, cell growth control, mitotic cell cycle, mitosis regulation, DNA replication	34
2—Cell differentiation & tissue development	Cell differentiation, angiogenesis, skeletal muscle tissue development, epidermis development, bone development, morphogenesis	17
3—Cell migration	(Regulation of) cell migration	8
4—Cell structure maintenance	Cytoskeleton organisation, microtubules structure, actin filament organisation, intermediate filament cytoskeleton organisation, mitochondrial genome maintenance, Golgi organisation, membrane structure, regulation of cell shape, blood vessel diameter maintenance, extracellular matrix assembly, cell adhesion	24
5—Cell aging & apoptosis	Aging, apoptosis, programmed cell death (regulation)	21
6—Signal transduction	Signal transduction, cell signalling (pathway)	15
7—Metabolism	Biosynthesis, anabolism, catabolism, transcription and translation regulation, glycolysis, proteolysis, protein degradation, protein homeostasis, protein folding, protein ubiquitination, phosphorylation, intracellular pH reduction, cell motility, neurotropic and neuroprotective activity, regulation of heart rate, muscle contraction, wound healing, protein secretion, regulation of the blood coagulation cascade	99
8—Transport	Intra- and extracellular transport, nuclear transport, ion and electron transport, protein transport, toxin transport, membrane fission, channel or molecule transporter	24
9—Stress response	Stress response, response to hydrogen peroxide, oxidative stress, hypoxia tolerance, response to xenobiotic stimulus, proteolytic stress response, heat stress response, response to increased oxygen levels, cellular hypotonic response, cell redox homeostasis	18
10—Immune response	Innate and adaptive immune response, allergic/inflammatory/antimicrobial response, cell response to interleukins (IL), regulation of macroautophagy	20
11—DNA repair	DNA repair	6

Note that because many proteins display more than one function the total number of proteins do not sum up to 162

## Results and discussion

Bufonids are worldwide distributed amphibians [1, 2] that store potent chemical weapon in their parotoid macroglands [6–8, 13]. Most of the studies dealing with toad parotoid secretions focus on biological activities of bufadienolides, biogenic amines and alkaloids [8, 11, 15]. Therefore, these molecules and their toxic and pharmacological effects are particularly well characterised [8]. Little is known about the protein and peptide content in the bufonid parotoids, as well as their toxic properties. Also, the processes related to poison production and excretion remain unknown.

Here, we identified 162 proteins in the extract from parotoid macroglands of *B. bufo* (Table 2; Additional file 1: Table A1). The most abundant (with the highest emPAI) were proteins related to the cell structure maintenance and proteins with enzymatic activities involved in metabolic pathways (see Additional file 1: Table A1). For each identified protein at least one biological function was assigned by searching UniProt database (Table 2). Then, all functions were assigned to one of 11 categories (Tables 1 and 2).

**Table 2:** Proteins identified in the extract from parotoids of the common toad *Bufo bufo* based on tandem mass spectrometry analysis

Accession code	Ion score			Mass [Da]			Matched peptides			Protein sequence coverage [%]			Protein name			Biological function					Function category
Q8VIF7	4539			52,958			76			6			Selenium binding protein 1			DNA replication, protein transport, response to hydrogen peroxide					1, 8, 9
Q4L0Y2	2490			41,986			61			37			Actin, cytoplasmic 1			Cell motility, transcription regulation					7
Q2I6W4	1425			59,993			38			13			Catalase			Aging, apoptosis regulation, cholesterol and haemoglobin metabolism					5, 7
Q4R5L2	1262			47,371			24			14			Alpha-enolase			Glycolysis, cell growth control, hypoxia tolerance, allergic responses					1, 7, 9, 10
P07323	630			47,433			9			8			Gamma-enolase			Neurotropic and neuroprotective activity, response to xenobiotic stimulus					7, 9
P13929	624			47,233			8			8			Beta-enolase			Glycolysis					7
P0C273	1019			14,949			23			31			Ubiquitin 60S ribosomal protein L40			DNA repair, cell cycle regulation, protein degradation, cell signalling					1, 6, 7, 11
A2Q0Z1	985			71,038			23			17			Heat shock cognate 71 kDa protein			Proteolytic stress response, cell cycle regulation					1, 9
P06761	85			72,440			3			3			78 kDa glucose regulated protein			Stress response, toxin transport, regulation of cell migration and apoptosis, cell response to IL-4					3, 5, 8–10
P62258	906			29,293			13			11			14–3-3 protein epsilon			Cell response to heat, mitotic cell cycle regulation					1, 9
Q5RAD2	841			16,827			21			41			Calmodulin			Calcium signal transduction pathway, cell cycle regulation, regulation of heart rate, cytokinesis regulation					1, 6, 7
Q2QD07	815			26,912			18			18			Triosephosphate isomerase			Glycolysis					7
A2Q0Z0	695			50,369			15			11			Elongation factor 1-alpha 1			Protein biosynthesis					7
Q5R7H8	658			5050			19			43			Thymosin beta-4			Cytoskeleton organisation, cell cycle regulation, cell migration, regulation of inflammatory response					1, 3, 4, 10
Q63610	599			29,173			15			18			Tropomyosin alpha-3 chain			Regulation of muscle contraction, cytoskeleton organisation					4, 7
Q5NVN0	454			58,395			10			7			Pyruvate kinase PKM			Glycolysis, regulation of cytoplasmic translation, regulation of programmed cell death					5, 7
Q3ZBT1	450			89,826			21			12			Transitional endoplasmic reticulum ATPase			Stress response, apoptosis, DNA repair					5, 9, 11
Q3MHM5	431			50,167			10			10			Tubulin beta-4B chain			Microtubules structure, mitotic cell cycle					1, 4
Q32L41	379			44,831			5			11			Phosphoglycerate kinase 1			Glycolysis, cell response to hypoxia, epithelial cell differentiation					2, 7, 9
Q32L41	336			9597			6			11			GTP cyclohydrolase 1 feedback regulatory protein			Phosphorylation					7
Q2PFW2	321			10,910			4			12			Small ubiquitin-related modifier 2			Nuclear transport, DNA replication, mitosis regulation, signal transduction					1, 6, 8
A5A6I5	312			39,777			4			3			Fructose-bisphosphate aldolase A			Glycolysis, regulation of cell migration					3, 7
Q1RMJ6	297			22,268			3			8			Rho-related GTP-binding protein RhoC			Signal transduction, cell migration, actin filament organisation, cell cycle cytokinesis					1, 3, 4, 6
Q8N6N7	293			9830			16			11			Acyl-CoA-binding domain-containing protein 7			Fatty acid metabolism					7
Q2PFL9	273			25,087			7			8			Peroxiredoxin-6			Cell protection against oxidative stress					9
Q5R5H2	258			68,554			5			4			V-type proton ATPase catalytic subunit A			Intracellular pH reduction, protein transmembrane transport, response to increased oxygen levels					7–9
O60218	252			36,181			6			2			Aldo–keto reductase family 1 member B10			Cellular detoxification of aldehyde					7
Q3SYU9	245			99,093			6			2			Major vault protein			Signal transduction, nucleo-cytoplasmic transport					6, 8
P28768	231			15,675			3			9			Superoxide dismutase [Mn], mitochondrial			Aging, apoptosis, cell response to oxidative stress					5, 9
P41361	223			52,728			5			3			Antithrombin-III			Regulation of the blood coagulation cascade					7
Q5R495	209			58,272			3			2			Serine/threonine protein kinase OSR1			Phosphorylation, cellular hypotonic response, intracellular signal transduction					6, 7, 9
Q4FZU2	209			59,489			4			1			Keratin, type II cytoskeletal 6A			Cell differentiation, wound healing, antimicrobial humoral immune response					2, 7, 10
Q3TXS7	208			106,490			3			1			26S proteasome non-ATPase regulatory subunit 1			Protein homeostasis, regulation of protein catabolism					7
Q4FZY0	208			26,743			3			5			EF-hand domain-containing protein D2			B-cell apoptosis control					5
Q4KMA2	208			43,516			3			7			UV excision repair protein RAD23 homolog B			DNA repair, cell response to IL-7					10, 11
P30838	206			50,685			6			5			Aldehyde dehydrogenase, dimeric NADP-preferring			Lipid metabolism, xenobiotic metabolism					7
Q9CR51	204			13,808			4			9			V-type proton ATPase subunit G 1			Cell response to increased oxygen levels					9
P19483	198			59,775			3			5			ATP synthase subunit alpha, mitochondrial			Aging, apoptosis, lipid metabolism, electron transport					5, 7, 8
P48644	191			55,276			4			3			Retinal dehydrogenase 1			Oxidoreductase activity					8
Q8HZM6	177			38,943			3			3			Annexin A1			Innate and adaptive immune response, actin cytoskeleton organisation					4, 10
P51857	175			37,629			9			9			3-oxo-5-beta-steroid 4-dehydrogenase			Bile acid biosynthesis					7
A6NEC2	174			54,127			4			4			Puromycin-sensitive aminopeptidase-like protein			Proteolysis					7
Q2HJ86	174			50,802			3			7			Tubulin alpha-1D chain			Cell division, cell response to IL-4, microtubule cytoskeleton organisation					1, 4, 10
Q3ZC84	173			52,990			3			3			Cytosolic non-specific dipeptidase			Proteolysis					7
C0HJG9	170			22,571			3			4			Annexin A2			Angiogenesis, heat stress response					2, 9
P10111	156			18,047			6			9			Peptidyl-prolyl cis–trans isomerase A			Apoptosis					5
P29117	44			22,072			2			8			Peptidyl-prolyl cis–trans isomerase F, mitochondrial			Apoptosis					5
Q3MHL4	153			48,021			4			6			Adenosylhomocysteinase			Chronic inflammatory response, response to hypoxia					9, 10
Q4R5L1	148			46,548			2			3			Aspartate aminotransferase, cytoplasmic			Biosynthesis of α-glutamate					7
Q4PLJ0	144			9066			5			17			NEDD8			Cell cycle control, proteolysis					1, 7
O46650	139			41,207			3			3			Alcohol dehydrogenase class-2 isozyme 2			Ethanol oxidation					7
Q3T0M7	137			23,836			7			9			Ran-specific GTPase-activating protein			Nucleo-cytoplasmatic transport					8
Q5E987	136			26,532			2			7			Proteasome subunit alpha type-5			Proteasomal protein catabolism					7
Q9JKB3	135			38,790			1			4			Y-box binding protein 3			Apoptosis					5
Q5E946	133			20,161			4			7			Protein deglycase DJ-1			DNA repair					11
Q2HWU2	126			57,590			4			1			Protein disulfide-isomerase			Cell response to IL-7					10
Q3THS6	120			43,937			4			11			S-adenosylmethionine synthase isoform type-2			S-adenosylmethionine biosynthesis					7
A3FKF7	116			35,981			3			4			Glyceraldehyde-3-phosphate dehydrogenase			Immune and antimicrobial response					10
Q3T0X5	112			29,797			3			6			Proteasome subunit alpha type-1			Proteolysis, immune response					7, 10
O35945	111			54,921			1			2			Aldehyde dehydrogenase, cytosolic 1			Ethanol metabolism					7
P00168	111			10,026			6			49			Cytochrome b5 (Fragment)			Electron transport					8
Q76KP1	108			116,855			7			1			N-acetyl-beta-glucosaminyl-glycoprotein 4-beta-N-acetylgalactosaminyltransferase 1			Transferase activity					7
P61458	106			12,024			4			29			Pterin-4-alpha-carbinolamine dehydratase			Tetrahydrobiopterin biosynthesis					7
Q5R844	99			17,057			3			8			Myosin light polypeptide 6			Muscle contraction, skeletal muscle tissue development					2, 7
Q15276	96			99,551			2			1			Rab GTPase-binding effector protein 1			Apoptosis, protein transport					5, 8
Q5R8F7	94			70,782			2			2			Polyadenylate-binding protein 1			mRNA processing, cell response to hypoxia					7, 9
P21836	89			68,447			4			1			Acetylcholinesterase			Neuronal apoptosis, cell adhesion					4, 5
Q9UBC2	85			94,289			2			1			Epidermal growth factor receptor substrate 15-like 1			Endosomal transport					8
P35908	83			65,623			1			1			Keratin, type II cytoskeletal 2 epidermal			Epidermis development					2
Q8HXW4	82			97,457			2			1			Glycogen phosphorylase, muscle form			Glucagon metabolism					7
Q52I78	73			55,569			2			4			Nicotinamide phosphoribosyltransferase			Signal transduction					6
Q9DAW9	72			36,544			2			6			Calponin-3			Regulation and metabolism of smooth muscle contraction					7
Q8VI73	71			37,500			2			4			Transaldolase			Carbohydrate metabolism					7
Q3SZD7	71			30,790			2			2			Carbonyl reductase [NADPH] 1			Epithelial cell differentiation					2
A0A1F3	68			36,892			1			3			L-lactate dehydrogenase A chain			Glycolysis					7
Q6IA69	68			80,291			4			1			Glutamine-dependent NAD( +) synthetase			NAD biosynthesis					7
O95340	68			69,916			1			2			Bifunctional 3'-phosphoadenosine 5'-phosphosulfate synthase 2			Blood coagulation, bone development, hormone metabolism, phosphorylation					2, 7
Q5RBE5	65			36,001			2			3			GDP-L-fucose synthase			Leukocyte cell–cell adhesion					4
Q4R4R7	64			35,069			1			4			Ribose-phosphate pyrophosphokinase 2			Nucleotide biosynthesis, phosphorylation					7
Q9Y2G7	63			63,081			8			3			Zinc finger protein 30 homolog			Transcription regulation					7
Q9CWM4	59			14,246			2			17			Prefoldin subunit 1			Protein folding and stabilisation					7
Q8JZP9	58			72,826			5			1			GAS2-like protein 1			Cytoskeleton structure					4
Q96CQ1	57			34,536			4			2			Solute carrier family 25			Mitochondrial genome maintenance					4
Q8IUE6	57			13,987			3			5			Histone H2A type 2B			Regulation of replication and transcription, DNA repair					1, 7, 11
Q4R4X6	57			23,669			1			5			Ras-related protein Rab-2A			Golgi organisation, intracellular protein transport					4, 8
Q2TBX6	55			26,413			1			3			Proteasome subunit beta type-1			Proteolysis					7
Q5XHY7	54			57,491			1			1			Signal transducing adapter molecule 2			Signal transduction, membrane fission					6
A6QLU8	54			48,623			5			1			Nucleoredoxin			Cell differentiation					2
Q9D1G2	54			22,132			2			4			Phosphomevalonate kinase			Cholesterol biosynthesis					7
Q0P5K3	53			17,173			1			7			Ubiquitin-conjugating enzyme E2			Protein ubiquitination					7
Q5BJP9	52			32,716			2			4			Phytanoyl-CoA dioxygenase domain-containing protein 1			Oxidoreductase activity					8
Q4R5E4	52			61,678			1			2			Phosphoglucomutase-1			Glycolysis					7
O00560	51			32,562			2			3			Syntenin-1			Actin cytoskeleton organisation, regulation of cell growth, migration and proliferation					1, 3, 4
Q5NVA2	49			55,315			1			1			Thioredoxin reductase 1, cytoplasmic			Cell population proliferation, signal transduction					1, 6
Q3ZCF3	49			18,784			1			7			S-phase kinase-associated protein 1			Cell cycle progression, signal transduction, transcription regulation, protein ubiquitination					1, 6, 7
O08782	48			36,547			2			2			Aldose reductase-related protein 2			Polyol metabolism, retinal metabolism					7
Q3U821	48			95,127			2			1			WD repeat-containing protein 75			rRNA processing					7
Q00915	47			15,974			1			6			Retinol-binding protein 1			Retinol transport in blood plasma					8
D3KU66	46			41,175			3			2			Acetylserotonin O-methyltransferase			Lipid metabolism, melatonin biosynthesis					7
Q8BK48	46			62,599			2			1			Pyrethroid hydrolase Ces2e			Prostaglandin metabolism					7
Q9CPU0	46			20,934			2			3			Lactoylglutathione lyase			Carbohydrate metabolism, regulation of transcription and apoptosis					5, 7
P02747	45			25,941			3			3			Complement C1q subcomponent subunit C			Immune response					10
A6QQV6	45			79,661			2			1			Protein arginine N-methyltransferase 7			Cell differentiation, regulation of protein binding					2, 7
Q8R3H9	44			44,679			2			1			Tetratricopeptide repeat protein 4			Innate immune response, protein folding					7, 10
Q68DL7	43			77,871			4			1			Uncharacterized protein C18orf63			Ubiquitin protein ligase activity					7
Q4R5E9	43			70,874			1			1			Secretogranin-2			Protein secretion, intracellular signal transduction, angiogenesis, endothelial cell migration, inflammatory response					2, 3, 6, 7, 10
A4Z6H0	43			50,343			2			3			Adenylosuccinate synthetase isozyme 1			AMP biosynthesis					7
Q5R8Y6	42			76,640			3			1			Transmembrane 9 superfamily member 2			Channel or small molecule transporter					8
Q6P6V1	41			69,685			3			1			Polypeptide N-acetylgalactosaminyltransferase 11			Signalling pathway					6
Q6ZQ82	41			92,610			2			1			Rho GTPase-activating protein 26			Actin cytoskeleton organisation, signal transduction					4, 6
Q8R238	40			35,078			1			4			Serine dehydratase-like			L-serine catabolism, lipid metabolism					7
Q2MHN2	40			21,389			1			5			Ferritin heavy chain			Iron transport, immune response, regulation of cell population proliferation					1, 8, 10
Q96QS6	39			43,137			1			2			Serine/threonine-protein kinase H2			Protein phosphorylation					7
Q6W3E5	39			72,363			1			1			Glycerophosphodiester phosphodiesterase domain-containing protein 4			Lipid metabolism					7
Q9BXB4	37			84,188			3			1			Oxysterol-binding protein-related protein 11			Lipid transport, fat cell differentiation					2, 8
Q7TQD2	37			23,698			1			5			Tubulin polymerization-promoting protein			Cell division, microtubule formation					1
Q4R362	37			11,360			1			9			Histone H4			DNA replication					1
O88879	37			142,798			2			1			Apoptotic protease-activating factor 1			Cell aging, apoptosis, cell differentiation					2, 5
Q8NEM1	37			63,275			1			1			Zinc finger protein 680			Transcription regulation					7
P97324	37			59,502			3			1			Glucose-6-phosphate 1-dehydrogenase 2			Oxidative stress, glucose metabolism, lipid metabolism					7, 9
Q0VGK2	37			59,722			4			2			Tetratricopeptide repeat protein 39C			Allium assembly, otolith morphogenesis					2
Q13410	37			59,383			3			2			Butyrophilin subfamily 1 member A1			Membrane structure					4
Q2EN76	36			17,257			1			11			Nucleoside diphosphate kinase B			Cell adhesion, GTP biosynthesis, transcription, apoptosis					4, 5, 7
Q9WVB0	36			21,848			5			4			RNA-binding protein with multiple splicing			Transcription, response to oxidative stress					7, 9
P48065	35			70,380			1			1			Sodium- and chloride-dependent betaine transporter			Amino acid transport					8
Q2M2U5	35			19,844			1			3			IQ domain-containing protein F2			Calmodulin binding activity					7
Q9BE72	35			67,948			1			2			Solute carrier family 2, facilitated glucose transporter member 12			Carbohydrate metabolism, glucose import					7
Q3SWX5	35			88,484			1			1			Cadherin-6			Cell–cell adhesion					4
Q8BHF7	34			62,818			6			1			CDP-diacylglycerol-glycerol-3-phosphate 3-phosphatidyltransferase, mitochondrial			Cardiolipin biosynthesis					7
Q4V8G5	34			41,325			2			2			Septin-12			Cell differentiation, protein localization					2, 7
Q4R4W5	34			26,669			1			3			Isopentenyl-diphosphate Delta-isomerase 1			Cholesterol biosynthesis					7
Q4FZT9	34			100,768			1			1			26S proteasome non-ATPase regulatory subunit 2			Cell cycle progression, apoptosis, DNA repair					1, 5, 11
Q9NQX1	34			74,699			2			1			PR domain zinc finger protein 5			Mitotic cell cycle, transcription regulation					1, 7
B7ZNG4	33			72,650			2			1			Tastin			Cell adhesion					4
Q9EP69	33			67,361			3			1			Phosphatidylinositide phosphatase SAC1			Phosphatidylinositol biosynthesis					7
Q8WUM4	32			96,469			1			1			Programmed cell death 6-interacting protein			Apoptosis, protein transport					5, 8
Q3SWU0	32			39,331			1			1			RISC-loading complex subunit TARBP2			Translation regulation, cell proliferation					1, 7
Q8TD55	32			53,593			2			1			Pleckstrin homology domain-containing family O member 2			Regulation of cell shape					4
A3KN27	32			60,420			2			2			Keratin, type II cytoskeletal 74			Intermediate filament cytoskeleton organisation					4
P48506	32			73,363			2			1			Glutamate-cysteine ligase catalytic subunit			Aging, cell redox homeostasis, blood vessel diameter maintenance					5, 7, 9
B2RZ78	32			20,594			1			5			Vacuolar protein sorting-associated protein 29			Intracellular protein transport					8
Q3T140	31			10,983			1			16			Dynein light chain roadblock-type 1			Microtubule-based movement					4
Q8K1R7	31			108,501			4			1			Serine/threonine protein kinase Nek9			Cell cycle, cell division, protein phosphorylation					1, 7
Q5NVP9	31			37,259			3			3			Mortality factor 4-like protein 1			Cell cycle, apoptosis, transcription regulation					1, 5, 7
Q9JI24	30			21,174			1			5			Interleukin-24			Apoptosis, cell response to IL-4 and LPS, regulation of cell migration					3, 5, 10
O35774	30			94,630			2			2			A-kinase anchor protein 4			Signal transduction, establishment of protein localisation					6, 7
Q9H7P6	30			35,919			1			3			Multivesicular body subunit 12B			Membrane fission, protein transport					8
Q8CG76	30			40,954			1			2			Aflatoxin B1 aldehyde reductase member 2			Lipid metabolism					7
A2VCK2	30			37,963			2			2			Doublecortin domain-containing protein 2B			Allium assembly, neuron migration					3
A4FUB0	30			73,548			1			1			Uncharacterized protein C5orf34 homolog			Regulation of calcium ion binding					8
Q9CXW2	30			41,258			2			3			28S ribosomal protein S22, mitochondrial			Mitochondrial translation					7
Q6IRU5	29			25,248			1			3			Clathrin light chain B			Intracellular protein transport					8
Q8NGL2	29			35,272			1			2			Olfactory receptor 5L1			G protein-coupled receptor signalling pathway					6
Q4KLM6	29			80,472			1			1			Prolyl 3-hydroxylase 2			Collagen metabolism, regulation of cell population proliferation					1, 7
Q9WUH5	29			56,607			2			2			Tripartite motif-containing protein 10			Erythrocyte differentiation, innate immune response					2, 10
O60336	29			165,188			1			1			Mitogen-activated protein kinase-binding protein 1			Regulation of antimicrobial response, regulation of IL-8 production, protein ubiquitination					7, 10
Q6IUU3	29			83,004			3			1			Sulfhydryl oxidase 1			Extracellular matrix assembly, protein folding, regulation of macroautophagy					4, 7, 10
Q28960	29			31,949			1			2			Carbonyl reductase [NADPH] 1			Epithelial cell differentiation, glucocorticoid metabolism, xenobiotic metabolism					2, 7
Q9BZB8	29			63,245			1			2			Cytoplasmic polyadenylation element-binding protein 1			Cell response to hypoxia					9
Q6P9L6	28			160,895			2			1			Kinesin-like protein KIF15			Mitotic cell cycle, microtubule-based movement					1, 4
Q53GT1	28			72,449			1			2			Kelch-like protein 22			Cell division, cell growth regulation					1
Q5RER6	26			38,650			1			3			Serine/threonine-protein kinase PDIK1L			Apoptosis, cell cycle, cell proliferation and differentiation, transcription regulation					1, 2, 5, 7

Function category: 1—Cell division & cell cycle regulation; 2—Cell differentiation & tissue development; 3—Cell migration; 4—Cell structure maintenance; 5—Cell aging & apoptosis; 6—Signal transduction; 7—Metabolism; 8—Transport; 9—Stress response; 10—Immune response; 11—DNA repair

STRING protein-protein interaction network predicted for proteins identified in the extract from *B. bufo* parotoids confirms the results obtained by searching the UniProt database (Additional file 2: Fig. A1). Most proteins are related to the metabolic processes with the strongest interactions between proteins involved in glycolysis, including phosphoglucomutase (pgm1), fructose-bisphosphate aldolase (aldoa), triosephosphate isomerase (tpi1), phosphoglycerate kinase 1 (pgk1) and transaldolase (taldo1). Strong interactions occur also between proteins, such as transitional endoplasmic reticulum ATPase (vcp), S-phase kinase-associated protein 1 (skp1), NEDD8 (nedd8), small ubiquitin-related modifier 2 (sumo2) and UV excision repair protein (rad23b), that participate in stress response and DNA repair and replication. This finding indicates that proteins present in the toad parotoids may effectively protect skin cells from DNA damage and thus minimize the harmful effects of UV radiation. Similarly, the results of GO enrichment and KEGG analyses are consistent with previous findings. Most predicted genes code intracellular proteins involved in metabolic processes and stress response (see Additional file 2: Figs A2–A5).

**Fig. 2:**
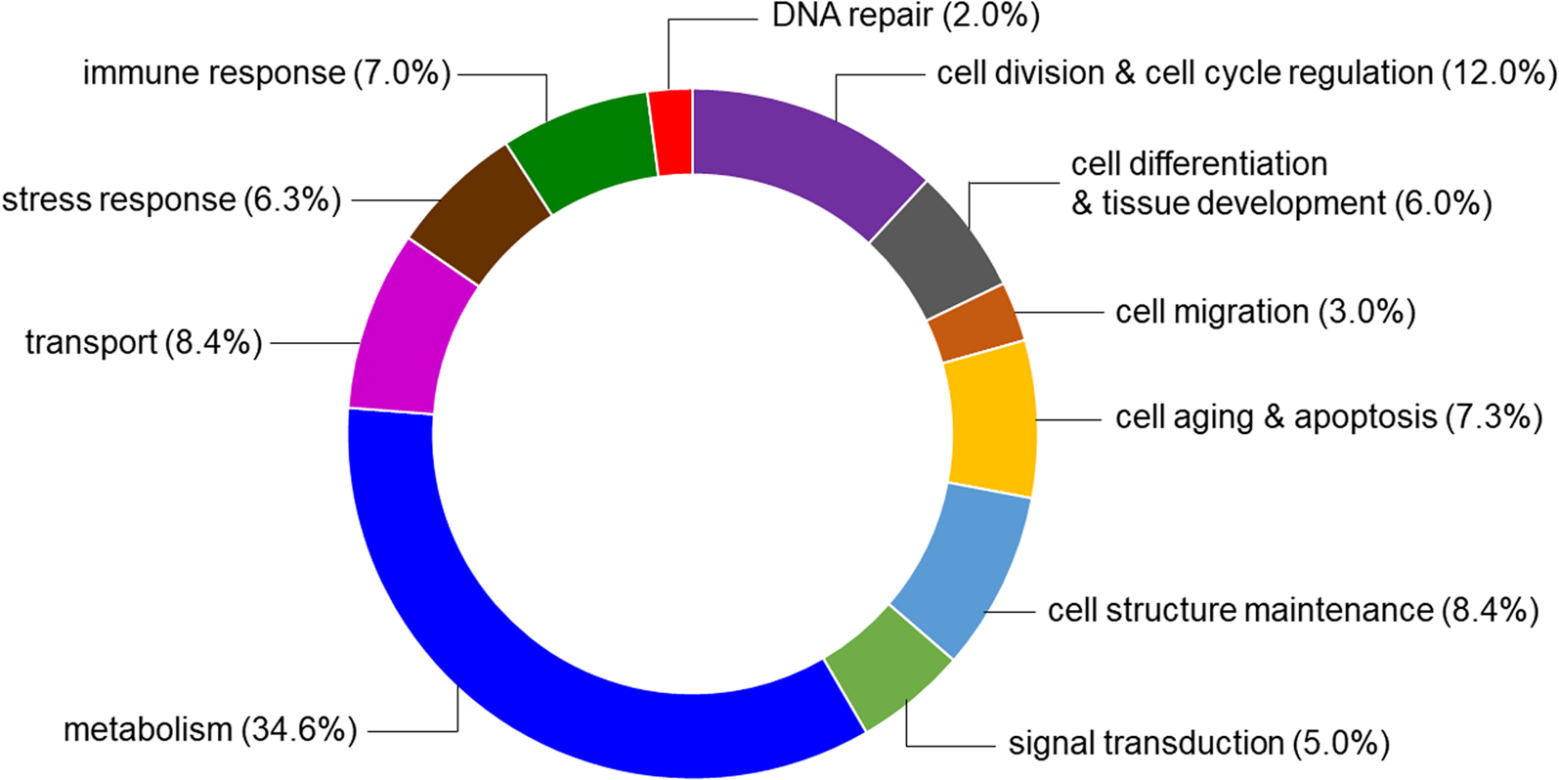
Biological functions of proteins identified in the extract from parotoids of the common toad *Bufo bufo*. Values in parentheses show percentage of proteins classified into each function category. See Table 2 for detailed functions of particular proteins

**Fig. 3:**
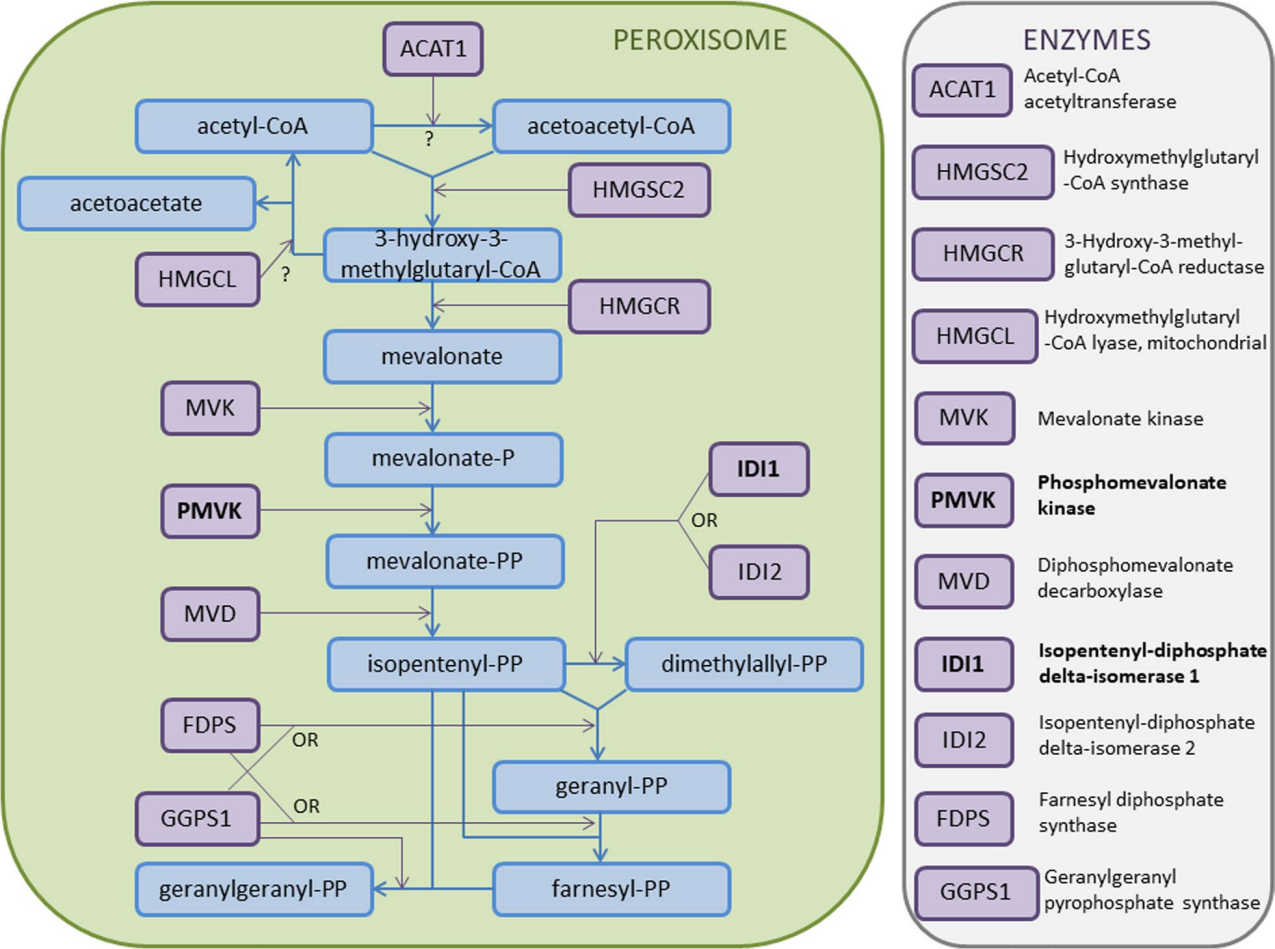
The mevalonate pathway in the cholesterol biosynthesis (according to Mazein et al. [27], modified). Blue arrows indicate synthesis processes, while purple arrows catalysis accelerated by enzymes. Question marks indicate uncertain processes. Enzymes (PMVK and IDI1) identified in this work in the extract from *B. bufo* parotoids are shown in bold

Regarding the number of identified proteins our results seem to be similar to those obtained recently by other researchers [e.g., 20, 23, 25] (see Introduction for details). One-third (34.6%) of the identified in this work proteins are involved in cell metabolism (Fig. 2 and Additional file 2: Figs A1–A4) with many of them being related to biosynthesis, regulation of transcription and translation, and gland functioning (Table 2 and Additional file 2: Figs A1–A4). This finding is consistent with the results of protein abundance analysis. Similarly, Sousa-Filho et al. [20] found numerous proteins related to cell metabolism and cell matrix in the *R. schneideri* parotoid secretion. Here, presence of two proteins, phosphomevalonate kinase (PMVK) and isopentenyl-diphosphate delta-isomerase 1 (IDI1; Table 2), is particularly noteworthy. These molecules are involved in the mevalonate arm of the cholesterol biosynthesis pathway [29, 30]. PMVK catalyses formation of mevalonate 5-diphosphate from mevalonate 5-phosphate, an essential step in the mevalonate pathway, while IDI1 performs isomerisation of isopentenyl diphosphate into dimethylallyl diphosphate (Fig. 3) [29, 30]. Cholesterol has been confirmed to be a precursor for synthesis of various steroids including bufadienolides [31–33], and it has been assumed that it is synthesised in the liver and then secreted and transferred through the bloodstream to the parotoid glands [31, 32]. Recent studies showed that genes coding PMVK and IDI1 are expressed in 27 human tissues including liver, kidney, spleen, adrenal glands and brain [34]. Genes coding IDI1 have been also proven to express in the liver of *X. tropicalis* frog [35]. PMVK has not been reported in any amphibian organs and tissues so far. Thus, confirming the presence of PMVK and IDI1 in the toad parotoid extract indicates that cholesterol necessary for toxin production may be also synthesised in parotoids, major glands that store bufadienolides.

Parotoids are holocrine glands [6, 36–38] in which poison excretion is accompanied by changes in the gland structure (Fig. 1B) [7]. Therefore, the complete poison replenishment is considered to be metabolically costly and time-consuming for toads [7], and may affect their growth and behaviour [39]. According to Jared et al. [7], the Cururu toad *Rhinella icterica* requires more than 3 months to restore the poison secreted from parotoids after mechanical compression of glands and the parotoid content seems to not return to the state preceding poison extraction. Rapid poison replenishment is necessary to ensure chemical protection against predators and microorganisms. Therefore, for toads, a reduced or depleted poison supply can be life-threatening because of diminished defence capabilities. In this work, we found various proteins involved in cell division and cell cycle regulation (12.0%), as well as cell aging and apoptosis (7.3%; Fig. 2). Together with many proteins that participate in molecule biosynthesis and transport (8.4%) as well as maintenance of cell structure (8.4%; Fig. 2), it may be indicative of a high epithelial cell turnover in parotoids. Such an intense cell turnover in the holocrine gland should require well developed protein machinery that regulates cell cycle and cell divisions to enable quick and effective replenishment of toxins and thus increase the chance of survival during encounter with a predator.

Some proteins found in this study are likely to reinforce toxic activity of parotoid secretion. For instance, gamma-enolase has neurotropic properties [40, 41] and thus its presence in parotoid secretion may increase paralytic activity of toad poison. Antithrombin-III regulates the blood coagulation cascade (Table 2) and may block blood clot formation [42, 43] and thus cause bleeding. Few proteins, such as puromycin-sensitive aminopeptidase-like protein, cytosolic non-specific dipeptidase or proteasome subunit alpha type-1, cause proteolysis [44–47] and thus may reinforce proteolytic action of toad poison. Previously, we also described in *B. bufo* parotoid secretion some peptides that may display physiological effects [13]. Mariano et al. [23] found proteasomes in *D. melanostictus* skin secretions. It is therefore likely that some molecules obtained in this work also participate in the toad chemical defence. However, further studies are required to confirm their biological activity and describe their action modes.

Tropomyosin alpha-3 chain, myosin light polypeptide 6 and calponin-3 are involved in regulation of muscle contraction (Table 2) [48–50]. Calmodulin is a Ca^+2^ binding protein that regulates apoptosis, inflammation and smooth muscle contraction [51–53]. Therefore, these proteins may promote poison excretion from parotoids. Also, 78 kDa glucose regulated protein, which is related to toxin transport [54], may participate in poison secretion from toad parotoids. Myosin light polypeptide 6 and calmodulin have already been reported in the *D. melanostictus* parotoid secretion [22, 23]. Calmodulin has been also found in the toad urinary bladder, oviduct and retina photoreceptors [55–57], while myosin and tropomyosin in smooth muscles within the carotid labyrinth of *Rh. marina* [58] and skeletal muscles of *X. tropicalis* and the Japanese tree frog *Hyla japonica* [59].

Mariano et al. [23] identified 62 binding proteins in the skin secretions of *D. melanostictus*. These proteins might bind to different molecules and play important role in amphibian skin secretion. In this work, we found acyl-CoA-binding domain-containing protein 7 and annexins in the *B. bufo* parotoid extract. The latter have been suggested to regulate diverse cellular and physiological processes, such as endo- and exocytosis [60, 61], and thus promote toxin excretion from poison glands. Both, acyl-CoA-binding proteins (ACBPs) and annexins, have recently been reported in *D. melanostictus* skin secretions [22, 23]. ACBPs have also been found in the frog’s brain [62], while annexins in the skin of the large-webbed bell toad *Bombina maxima*, the Dybovsky’s frog *Rana dybowskii*, and the Chinese giant salamander *Andrias davidianus* [63–65]. It seems that the latter are unique to the skin and because of their antimicrobial activity related to the immune response of amphibian skin secretions.

It is well known that amphibian skin plays an important role in antimicrobial defence [66, 67]. Proteins related to immune defence have already been reported in anuran skin secretions [18, 66–68]. Also, in this work we found proteins involved in innate and adaptive immune response, and antimicrobial, allergic and inflammatory response (7.0%; Table 2, Fig. 2). Thus, our results confirm antimicrobial functions of bufonid skin secretions. Also, proteins related to stress response are quite abundant (6.3%) in the extract from *B. bufo* parotoids (Fig. 2 and Additional file 2: Figs A1–A3). Here, we identified catalase, peroxiredoxin 6 and superoxide dismutase that are related to the anti-oxidant system [69]. Similar proteins (catalase, glutathione peroxidase 3, peroxiredoxin 6, superoxide dismutase and thioredoxin like 1) have previously been reported in parotoid secretion of *R. schneideri* [20]. Superoxide dismutase has also been identified in skin secretions of *R. dybowskii* [64], while catalase in lungs, heart, liver and kidney of the painted frog *Discoglossus pictus* [70].

Amphibian populations are in decline, which has been exacerbated in the last few decades and caused by several factors [71]. Ultraviolet (UV) radiation is especially dangerous to amphibians because of their thin and poorly cornified integument [72], and may lead to protein denaturation, cell damage and death, and/or mutagenesis in amphibian skin [73, 74]. Amphibians can cope with the harmful effects of UV radiation in many ways which include behavioural and molecular mechanisms. Here, we report 6 proteins in the extract from toad parotoids (Table 2, Fig. 2 and Additional file 2: Fig. A1) that are involved in DNA repair. Also, heat shock proteins (HSPs) may be synthesised under environmental or physiological stress [75]. They interact with denatured proteins and help them to refold and reassemble, turning back their active forms [23, 76]. Therefore, all these molecules may represent molecular mechanisms that may effectively protect toad skin cells from DNA damage and thus minimize the harmful effects of UV radiation. Such proteins involved in protection against UV radiation damage and protein recycling have been recently found in the skin secretion of *D. melanostictus* [23]. Related to stress response HSPs have been reported for many anuran organs, such as skin, liver, heart, kidneys, lungs and brain [77, 78].

## Conclusions

We report here housekeeping proteins in the extract from *B. bufo* parotoid macroglands. Applying a proteomic approach enabled us to obtain new findings on the molecules that are involved in poison production and secretion, and may contribute to the gland functioning. Most identified proteins are involved in metabolic processes and cell structure maintenance. Our results indicate that in toads cholesterol may be synthesized in parotoids, and not only in the liver from which is then transferred through the bloodstream to the parotoids. Presence of proteins that protect skin cells from DNA damage may help to minimize the harmful effects of UV radiation. It opens up new perspectives for studying biological activity of molecules from parotoid secretions and their role in the toad chemical defence system. Applying de novo peptide sequencing coupled with analyses of various databases will provide new protein dataset in anuran skin secretions and extend our knowledge on the functioning of bufonid parotoids.

## Supplementary Information

**Additional file 1**. **Table A1.** Peptide sequences identified in the extract from parotoids of the common toad *Bufo bufo* based on tandem mass spectrometry analysis

**Additional file 2**. **Figures A1-A5. Fig. A1** STRING protein-protein interaction network predicted for proteins identified in the extract from parotoids of the common toad *Bufo bufo* based on homology to *Xenopus tropicalis*. Red balls represent proteins involved in metabolic processes, while lines show interactions between proteins. The thicker the line, the stronger the interaction. Abbreviations within the balls represent protein labels. Full protein names and their labels are provided in Table A1 in Additional file 1. **Fig. A2** Number of proteins involved in metabolic processes predicted based on the Gene ontology enrichment analysis of proteins identified in the extract from parotoids of the common toad *Bufo bufo*. **Fig. A3** Molecular functions and number of proteins predicted based on the Gene ontology enrichment analysis of proteins identified in the extract from parotoids of the common toad *Bufo bufo*. **Fig. A4** Number of proteins involved in signalling pathways predicted based on the KEGG analysis of proteins identified in the extract from parotoids of the common toad *Bufo bufo*. **Fig. A5** Localisation of proteins identified in the extract from parotoids of the common toad *Bufo bufo* within the cell based on the Gene ontology component analysis

## Data Availability

All data are available in the main text and the supplementary information files. Further information and requests for data should be directed to and will be fulfilled by the corresponding author.
