# Supplementary material for: Proteins from toad’s parotoid macroglands: do they play a role in gland functioning and chemical defence?
Source: Front Zool. 2023 Jun 16;20:21. doi: 10.1186/s12983-023-00499-8 (PMC10273630; doi:10.1186/s12983-023-00499-8)
Supplement: Supplementary file 1 — Additional file 1. Table A1. Peptide sequences identified in the extract from parotoids of the common toad Bufo bufo based on tandem mass spectrometry analysis [file 12983_2023_499_MOESM1_ESM.docx]

**Proteins from toad’s parotoid macroglands: Do they play a role in gland functioning and chemical defence?**

Krzysztof Kowalski^1*^, Paweł Marciniak^2^ and Leszek Rychlik^3^

^1^Department of Vertebrate Zoology and Ecology, Institute of Biology, Faculty of Biological and Veterinary Sciences, Nicolaus Copernicus University, Lwowska 1, Toruń, 87-100, Poland, e-mail: k.kowalski@umk.pl,: tel. +48 56 611 4910

^2^Department of Animal Physiology and Developmental Biology, Institute of Experimental Biology, Faculty of Biology, Adam Mickiewicz University, Uniwersytetu Poznańskiego 6, Poznań, 61-614, Poland, e-mail: pmarcin@amu.edu.pl, tel.: +48 61 829 5926

^3^Department of Systematic Zoology, Institute of Environmental Biology, Faculty of Biology, Adam Mickiewicz University, Uniwersytetu Poznańskiego 6, Poznań, 61-614, Poland, e-mail: leszek.rychlik@amu.edu.pl, tel.: +48 61 829 5751

*Correspondence: k.kowalski@umk.pl (K.K.)

**Table A1** Peptide sequences identified in the extract from parotoids of the common toad *Bufo bufo* based on tandem mass spectrometry analysis

| **Accession code** | **Ion Score** | **Mass [Da]** | **Matched peptides** | **Protein sequence coverage [%]** | **emPAI** | **Identified peptides** | **Protein name** | **Protein label** |
| --- | --- | --- | --- | --- | --- | --- | --- | --- |
| Q8VIF7 | 4539 | 52958 | 76 | 6 | 0.49 | K.RVPGGPQMIQLSLDGK.R  K.RVPGGPQMIQLSLDGK.R+ Oxidation (M)  R.VPGGPQMIQLSLDGK.R  R.VPGGPQMIQLSLDGK.R+ Oxidation (M)  R.VPGGPQMIQLSLDGKR.L  R.VPGGPQMIQLSLDGKR.L+ Oxidation (M)  R.YPGGDCSSDIWI.- | Selenium binding protein 1 | selenbp1 |
| Q4L0Y2 | 2490 | 41986 | 61 | 37 | 3.50 | K.AGFAGDDAPR.A  R.AVFPSIVGRPR.H  R.HQGVMVGMGQK.D  R.HQGVMVGMGQK.D+ Oxidation (M)  R.HQGVMVGMGQK.D+ 2 Oxidation (M)  K.DSYVGDEAQSK.R  K.DSYVGDEAQSKR.G  K.IWHHTFYNELR.V  R.VAPEEHPVLLTEAPLNPK.A  R.TTGIVMDSGDGVTHTVPIYEGYALPHAILR.L  R.TTGIVMDSGDGVTHTVPIYEGYALPHAILR.L+ Oxidation (M)  R.GYSFTTTAER.E  K.SYELPDGQVITIGNER.F  K.EITALAPSTMK.I  K.EITALAPSTMK.I+ Oxidation (M) | Actin, cytoplasmic 1 | actb |
| Q2I6W4 | 1425 | 59993 | 38 | 13 | 0.88 | R.GPLLVQDVVFTDEMAHFDR.E  R.GPLLVQDVVFTDEMAHFDR.E+ Oxidation (M)  R.GPLLVQDVVFTDEMAHFDRER.I  R.GPLLVQDVVFTDEMAHFDRER.I+ Oxidation (M)  K.GAGAFGYFEVTHDITKYSK.A  R.GIPDGHR.H  R.LFSYPDTHR.H  R.VANYQR.D  K.DAQLFIQK.K | Catalase | cat |
| Q4R5L2 | 1262 | 47371 | 24 | 14 | 0.70 | R.EIFDSR.G  R.AAVPSGASTGIYEALELR.D  K.IDKLMIEMDGTENK.S  K.LMIEMDGTENK.S  K.LMIEMDGTENK.S+ Oxidation (M)  R.IGAEVYHNLK.N  K.YNQLLR.I  R.IEEELGSK.A | Alpha-enolase | eno1 |
| P07323 | 630 | 47433 | 9 | 8 | 0.31 | R.AAVPSGASTGIYEALELR.D  K.LDNLMLELDGTENK.S+ Oxidation (M)  K.YNQLMR.I | Gamma-enolase | eno2 |
| P13929 | 624 | 47233 | 8 | 8 | 0.22 | R.AAVPSGASTGIYEALELR.D  K.FMIELDGTENK.S  K.YNQLMR.I | Beta-enolase | eno3 |
| P0C273 | 1019 | 14949 | 23 | 31 | 2.97 | -.MQIFVK.T  K.TITLEVEPSDTIENVK.A  R.TLSDYNIQK.E  R.TLSDYNIQKESTLHLVLR.L  K.ESTLHLVLR.L | Ubiquitin 60S ribosomal protein L40 | uba52 |
| A2Q0Z1 | 985 | 71038 | 23 | 17 | 0.61 | K.VEIIANDQGNR.T  R.TTPSYVAFTDTER.L  R.IINEPTAAAIAYGLDKK.V  K.STAGDTHLGGEDFDNR.M  R.ARFEELNADLFR.G  K.LDKSQIHDIVLVGGSTR.I  K.SQIHDIVLVGGSTR.I  K.QTQTFTTYSDNQPGVLIQVYEGER.A | Heat shock cognate 71 kDa protein | hspa8 |
| P06761 | 85 | 72440 | 3 | 3 | 0.12 | R.VEIIANDQGNR.I  K.KSDIDEIVLVGGSTR.I | 78 kDa glucose regulated protein | hspa5 |
| P62258 | 906 | 29293 | 13 | 11 | 0.77 | R.EDLVYQAK.L  K.VAGMDVELTVEER.N  K.VAGMDVELTVEER.N+ Oxidation (M)  R.NLLSVAYK.N | 14-3-3 protein epsilon | ywhae |
| Q5RAD2 | 841 | 16827 | 21 | 41 | 3.37 | K.EAFSLFDKDGDGTITTK.E  K.DGDGTITTK.E  K.ELGTVMR.S  R.VFDKDGNGYISAAELR.H  R.EADIDGDGQVNYEEFVQMMTAK.-+ Oxidation (M)  R.EADIDGDGQVNYEEFVQMMTAK.-+ 2 Oxidation (M) | Calmodulin | calm |
| Q2QD07 | 815 | 26912 | 18 | 18 | 1.18 | R.KFFVGGNWK.M  R.RHVFGESDELIGQK.V  R.HVFGESDELIGQK.V  K.VVFEQTK.V  K.VVLAYEPVWAIGTGK.T | Triosephosphate isomerase | tpi1 |
| A2Q0Z0 | 695 | 50369 | 15 | 11 | 0.52 | R.LPLQDVYK.I  K.IGGIGTVPVGR.V  R.VETGVLKPGMVVTFAPVNVTTEVK.S  R.VETGVLKPGMVVTFAPVNVTTEVK.S+ Oxidation (M)  R.QTVAVGVIK.A | Elongation factor 1-alpha 1 | eef1a1 |
| Q5R7H8 | 658 | 5050 | 19 | 43 | 22.34 | K.TETQEK.N  K.TETQEKNPLPSK.E  K.NPLPSKETIEQEK.Q  K.ETIEQEK.Q | Thymosin beta-4 | tmsb4 |
| Q63610 | 599 | 29173 | 15 | 18 | 0.78 | R.KIQVLQQQADDAEER.A  R.IQLVEEELDR.A  R.AQERLATALQK.L  R.KLVIIEGDLER.T  K.LVIIEGDLER.T | Tropomyosin alpha-3 chain | tpm3 |
| Q5NVN0 | 454 | 58395 | 10 | 7 | 0.33 | R.NTGIICTIGPASR.S  K.GSGTAEVELK.K  K.GDYPLEAVR.M  R.APIIAVTR.N | Pyruvate kinase PKM | pkm |
| Q3ZBT1 | 450 | 89826 | 21 | 12 | 0.53 | R.AHVIVMAATNRPNSIDPALR.R  R.AHVIVMAATNRPNSIDPALR.R+ Oxidation (M)  R.AHVIVMAATNRPNSIDPALRR.F  R.EVDIGIPDATGR.L  R.LEILQIHTK.N  R.WALSQSNPSALR.E  R.ETVVEVPQVTWEDIGGLEDVKR.E  R.RDHFEEAMR.F  R.KYEMFAQTLQQSR.G+ Oxidation (M) | Transitional endoplasmic reticulum ATPase | vcp |
| Q3MHM5 | 431 | 50167 | 10 | 10 | 0.40 | K.FWEVISDEHGIDPTGTYHGDSDLQLER.I  R.YLTVAAVFR.G  R.ISEQFTAMFR.R  R.ISEQFTAMFR.R+ Oxidation (M) | Tubulin beta-4B chain | tubb4b |
| Q3T0P6 | 379 | 44831 | 5 | 4 | 0.10 | K.VSHVSTGGGASLELLEGK.V | Phosphoglycerate kinase 1 | pgk1 |
| Q32L41 | 336 | 9597 | 6 | 11 | 0.53 | M.PYLLISTQIR.M | GTP cyclohydrolase 1 feedback regulatory protein | gchfr |
| Q2PFW2 | 321 | 10910 | 4 | 12 | 0.46 | K.VAGQDGSVVQFK.I | Small ubiquitin-related modifier 2 | sumo2 |
| A5A6I5 | 312 | 39777 | 4 | 3 | 0.11 | K.GILAADESTGSIAK.R | Fructose-bisphosphate aldolase A | aldoa |
| Q1RMJ6 | 297 | 22268 | 3 | 8 | 0.21 | K.QVELALWDTAGQEDYDR.L | Rho-related GTP-binding protein RhoC | rhoc |
| Q8N6N7 | 293 | 9830 | 16 | 11 | 2.45 | K.AKWEAWNLK.K  K.WEAWNLK.K  K.WEAWNLKK.G | Acyl-CoA-binding domain-containing protein 7 | acbd7 |
| Q2PFL9 | 273 | 25087 | 7 | 8 | 0.40 | K.LSILYPATTGR.N  R.NFDEILR.V | Peroxiredoxin-6 | prdx6 |
| Q5R5H2 | 258 | 68554 | 5 | 4 | 0.13 | R.DMGYHVSMMADSTSR.W  K.ASLAETDKITLEVAK.L | V-type proton ATPase catalytic subunit A | atp6v1a |
| O60218 | 252 | 36181 | 6 | 2 | 0.12 | K.TAAQVLIR.F | Aldo-keto reductase family 1 member B10 | akr1b10 |
| Q3SYU9 | 245 | 99093 | 6 | 2 | 0.09 | R.VPHNAAVQVYDYR.E  R.IEGEGAVLQAK.L | Major vault protein | mvp |
| P28768 | 231 | 15675 | 3 | 9 | 0.30 | K.GDVTAQVALQPALK.F | Superoxide dismutase [Mn], mitochondrial | sod2 |
| P41361 | 223 | 52728 | 5 | 3 | 0.17 | R.VWELSK.A  R.FRIEDSFSVK.E | Antithrombin-III | serpinc1 |
| Q5R495 | 209 | 58272 | 3 | 2 | 0.08 | R.DLVIVAANLQK.I | Serine/threonine protein kinase OSR1 | oxsr1 |
| Q4FZU2 | 209 | 59489 | 4 | 1 | 0.07 | K.YEELQITAGR.H | Keratin, type II cytoskeletal 6A | krt6a |
| Q3TXS7 | 208 | 106490 | 3 | 1 | 0.04 | K.VSTAVLSITAK.A | 26S proteasome non-ATPase regulatory subunit 1 | psmd1 |
| Q4FZY0 | 208 | 26743 | 3 | 5 | 0.17 | R.LSEIDVSTEGVK.G | EF-hand domain-containing protein D2 | efhd2 |
| Q4KMA2 | 208 | 43516 | 3 | 7 | 0.21 | K.NFVVVMVTKPK.A  R.QIIQQNPSLLPALLQQIGR.E | UV excision repair protein RAD23 homolog B | rad23b |
| P30838 | 206 | 50685 | 6 | 5 | 0.28 | R.TRPLQFR.I  K.IIMTAAAK.H  K.HLTPVTLELGGK.S | Aldehyde dehydrogenase, dimeric NADP-preferring | aldh3a1 |
| Q9CR51 | 204 | 13808 | 4 | 9 | 0.35 | K.EEAQAEIEQYR.L | V-type proton ATPase subunit G 1 | atp6v1g1 |
| P19483 | 198 | 59775 | 3 | 5 | 0.15 | K.TGTAEVSSILEER.I  R.NVQAEEMVEFSSGLK.G+ Oxidation (M) | ATP synthase subunit alpha, mitochondrial | atp5a1 |
| P48644 | 191 | 55276 | 4 | 3 | 0.16 | K.VAFTGSTEVGK.L  R.VSLELGGK.S | Retinal dehydrogenase 1 | aldh1a1 |
| Q8HZM6 | 177 | 38943 | 3 | 3 | 0.11 | K.DITSDTSGDFQK.A | Annexin A1 | anxa1 |
| P51857 | 175 | 37629 | 9 | 9 | 0.40 | K.SLGVSNFNR.R  R.RQLELILNKPGLK.H  K.TAAQIVLR.F | 3-oxo-5-beta-steroid 4-dehydrogenase | akr1d1 |
| A6NEC2 | 174 | 54127 | 4 | 4 | 0.17 | R.YAAVTQFEATDAR.R  K.FALEVAAK.T | Puromycin-sensitive aminopeptidase-like protein | npeppsl1 |
| Q2HJ86 | 174 | 50802 | 3 | 7 | 0.18 | R.AVFVDLEPTVIDEVR.T  R.AFVHWYVGEGMEEGEFSEAR.E+ Oxidation (M) | Tubulin alpha-1D chain | tuba1d |
| Q3ZC84 | 173 | 52990 | 3 | 3 | 0.08 | R.EGGSIPVTLTFQEATGK.N | Cytosolic non-specific dipeptidase | cndp2 |
| C0HJG9 | 170 | 22571 | 3 | 4 | 0.20 | R.QDIAFAYQR.K | Annexin A2 | anxa2 |
| P10111 | 156 | 18047 | 6 | 9 | 0.58 | K.TAENFR.A  K.KITISDCGQL.- | Peptidyl-prolyl cis-trans isomerase A | ppia |
| P29117 | 44 | 22072 | 2 | 8 | 0.46 | K.TAENFR.A  K.GFGYKGSTFHR.V | Peptidyl-prolyl cis-trans isomerase F, mitochondrial | ppif |
| Q3MHL4 | 153 | 48021 | 4 | 6 | 0.19 | R.GISEETTTGVHNLYK.M  K.KLDEAVAEAHLGK.L | Adenosylhomocysteinase | ahcy |
| Q4R5L1 | 148 | 46548 | 2 | 3 | 0.09 | R.VGGVQSLGGTGALR.I | Aspartate aminotransferase, cytoplasmic | got1 |
| Q4PLJ0 | 144 | 9066 | 5 | 17 | 0.56 | K.EIEIDIEPTDKVER.I | NEDD8 | nedd8 |
| O46650 | 139 | 41207 | 3 | 3 | 0.23 | R.IIAVDINSDK.F  R.IIAVDINSDKFAK.A | Alcohol dehydrogenase class-2 isozyme 2 | adh2 |
| Q3T0M7 | 137 | 23836 | 7 | 9 | 0.19 | K.TLEEDEEELFK.M  R.KEIEEK.E  K.EIEEKEK.K | Ran-specific GTPase-activating protein | ranbp1 |
| Q5E987 | 136 | 26532 | 2 | 7 | 0.17 | R.AIGSASEGAQSSLQEVYHK.S | Proteasome subunit alpha type-5 | psma5 |
| Q9JKB3 | 135 | 38790 | 1 | 4 | 0.11 | R.SVGDGETVEFDVVEGEK.G | Y-box binding protein 3 | ybx3 |
| Q5E946 | 133 | 20161 | 4 | 7 | 0.51 | R.ALVILAK.G  K.APLVLKD.- | Protein deglycase DJ-1 | park7 |
| Q2HWU2 | 126 | 57590 | 4 | 1 | 0.16 | R.LITLEEEMTK.Y  R.LITLEEEMTK.Y+ Oxidation (M) | Protein disulfide-isomerase | p4hb |
| Q3THS6 | 120 | 43937 | 4 | 11 | 0.47 | K.TGMILLAGEITSR.A+ Oxidation (M)  K.TQVTVQYMQDR.G  K.TQVTVQYMQDR.G+ Oxidation (M)  R.KIIVDTYGGWGAHGGGAFSGK.D | S-adenosylmethionine synthase isoform type-2 | mat2a |
| A3FKF7 | 116 | 35981 | 3 | 4 | 0.12 | R.GAAQNIIPASTGAAK.A | Glyceraldehyde-3-phosphate dehydrogenase | gapdh |
| Q3T0X5 | 112 | 29797 | 3 | 6 | 0.33 | R.LVSLIGSK.T  K.NVSIGIVGK.D | Proteasome subunit alpha type-1 | psma1 |
| O35945 | 111 | 54921 | 1 | 2 | 0.08 | R.ANNTTYGLAAGVFTK.D | Aldehyde dehydrogenase, cytosolic 1 | aldh1a7 |
| P00168 | 111 | 10026 | 6 | 49 | 2.41 | K.YYTLEEIQK.H  K.FLEEHPGGEEVLR.E  R.EQAGGDATEDFEDVGHSTDAR.E | Cytochrome b5 (Fragment) | cyb5a |
| Q76KP1 | 108 | 116855 | 7 | 1 | 0.04 | R.QDVMVHFIVPVK.N | N-acetyl-beta-glucosaminyl-glycoprotein 4-beta-N-acetylgalactosaminyltransferase 1 | b4galnt4 |
| P61458 | 106 | 12024 | 4 | 29 | 1.8 | R.DQLLPNLR.A  R.AVGWNEVEGR.D  K.LDHHPEWFNVYNK.V | Pterin-4-alpha-carbinolamine dehydratase | pcbd1 |
| Q5R844 | 99 | 17057 | 3 | 8 | 0.27 | K.LDHHPEWFNVYNK.V | Myosin light polypeptide 6 | myl6 |
| Q15276 | 96 | 99551 | 2 | 1 | 0.04 | K.LSQTLQVQLER.I | Rab GTPase-binding effector protein 1 | rabep1 |
| Q5R8F7 | 94 | 70782 | 2 | 2 | 0.06 | R.SKVDEAVAVLQAHQAK.E | Polyadenylate-binding protein 1 | pabpc1 |
| P21836 | 89 | 68447 | 4 | 1 | 0.06 | K.YWTNFAR.T | Acetylcholinesterase | ache |
| Q9UBC2 | 85 | 94289 | 2 | 1 | 0.05 | K.SGLSDIILGK.I | Epidermal growth factor receptor substrate 15-like 1 | eps15l1 |
| P35908 | 83 | 65623 | 1 | 1 | 0.07 | K.YEELQVTVGR.H | Keratin, type II cytoskeletal 2 epidermal | krt2 |
| Q8HXW4 | 82 | 97457 | 2 | 1 | 0.04 | R.HLQIIYEINQR.F | Glycogen phosphorylase, muscle form | pygm |
| Q52I78 | 73 | 55569 | 2 | 4 | 0.08 | R.GVSSQETAGIGASAHLVNFK.G | Nicotinamide phosphoribosyltransferase | nampt |
| Q9DAW9 | 72 | 36544 | 2 | 6 | 0.12 | K.MQTDKPFDQTTISLQMGTNK.G+ Oxidation (M) | Calponin-3 | cnn3 |
| Q8VI73 | 71 | 37500 | 2 | 4 | 0.25 | R.VSTEVDAR.L  K.FAADAIK.L | Transaldolase | taldo1 |
| Q3SZD7 | 71 | 30790 | 2 | 2 | 0.15 | K.IGVTVLSR.I | Carbonyl reductase [NADPH] 1 | cbr1 |
| A0A1F3 | 68 | 36892 | 1 | 3 | 0.12 | K.KSADTLWGIQK.E | L-lactate dehydrogenase A chain | ldha |
| Q6IA69 | 68 | 80291 | 4 | 1 | 0.05 | R.VDLVTMVTSK.N+ Oxidation (M) | Glutamine-dependent NAD(+) synthetase | nadsyn1 |
| O95340 | 68 | 69916 | 1 | 2 | 0.06 | K.EMNADAVFAFQLR.N+ Oxidation (M) | Bifunctional 3'-phosphoadenosine  5'-phosphosulfate synthase 2 | papss2 |
| Q5RBE5 | 65 | 36001 | 2 | 3 | 0.12 | R.ILVTGGSGLVGK.A | GDP-L-fucose synthase | tsta3 |
| Q4R4R7 | 64 | 35069 | 1 | 4 | 0.13 | K.FSNQETSVEIGESVR.G | Ribose-phosphate pyrophosphokinase 2 | prps2 |
| Q9Y2G7 | 63 | 63081 | 8 | 3 | 0.07 | K.EPWMVVRDEK.R+ Oxidation (M)  K.IFTCGSDLR.V | Zinc finger protein 30 homolog | zfp30 |
| Q9CWM4 | 59 | 14246 | 2 | 17 | 0.34 | -.MAASVDLELK.K  K.LADIQIEQLNR.T | Prefoldin subunit 1 | pfdn1 |
| Q8JZP9 | 58 | 72826 | 5 | 1 | 0.06 | R.GGGGSGGSGR.S | GAS2-like protein 1 | gas2l1 |
| Q96CQ1 | 57 | 34536 | 4 | 2 | 0.13 | K.VILEKEGPR.S | Solute carrier family 25 | slc25a36 |
| Q8IUE6 | 57 | 13987 | 3 | 5 | 0.34 | R.HLQLAVR.N | Histone H2A type 2B | hist2h2ab |
| Q4R4X6 | 57 | 23669 | 1 | 5 | 0.19 | R.EHGLIFMETSAK.T | Ras-related protein Rab-2A | rab2a |
| Q2TBX6 | 55 | 26413 | 1 | 3 | 0.17 | K.DVFISAAER.D | Proteasome subunit beta type-1 | psmb1 |
| Q5XHY7 | 54 | 57491 | 1 | 1 | 0.08 | K.AIELSLQEQK.Q | Signal transducing adapter molecule 2 | stam2 |
| A6QLU8 | 54 | 48623 | 5 | 1 | 0.09 | K.IKEAGQK.F | Nucleoredoxin | nxn |
| Q9D1G2 | 54 | 22132 | 2 | 4 | 0.21 | R.LVLLFSGK.R | Phosphomevalonate kinase | pmvk |
| Q0P5K3 | 53 | 17173 | 1 | 7 | 0.27 | K.TNEAQAIETAR.A | Ubiquitin-conjugating enzyme E2 | ube2n |
| Q5BJP9 | 52 | 32716 | 2 | 4 | 0.14 | R.GGLVLIHGEVVHK.S | Phytanoyl-CoA dioxygenase domain-containing protein 1 | phyhd1 |
| Q4R5E4 | 52 | 61678 | 1 | 2 | 0.07 | R.LSGTGSAGATIR.L | Phosphoglucomutase-1 | pgm1 |
| O00560 | 51 | 32562 | 2 | 3 | 0.14 | K.DSTGHVGFIFK.N | Syntenin-1 | sdcbp |
| Q5NVA2 | 49 | 55315 | 1 | 1 | 0.08 | K.IICNTK.D | Thioredoxin reductase 1, cytoplasmic | txnrd1 |
| Q3ZCF3 | 49 | 18784 | 1 | 7 | 0.25 | K.NDFTEEEEAQVR.K | S-phase kinase-associated protein 1 | skp1 |
| O08782 | 48 | 36547 | 2 | 2 | 0.12 | R.NVVVIPK.S | Aldose reductase-related protein 2 | akr1b8 |
| Q3U821 | 48 | 95127 | 2 | 1 | 0.05 | R.RAVFSVDSK.Y | WD repeat-containing protein 75 | wdr75 |
| Q00915 | 47 | 15974 | 1 | 6 | 0.30 | R.ALDVNVALR.K | Retinol-binding protein 1 | rbp1 |
| D3KU66 | 46 | 41175 | 3 | 2 | 0.11 | R.VLTAFDLSPFR.V | Acetylserotonin O-methyltransferase | asmt |
| Q8BK48 | 46 | 62599 | 2 | 1 | 0.07 | K.HPQELMASK.D+ Oxidation (M) | Pyrethroid hydrolase Ces2e | ces2e |
| Q9CPU0 | 46 | 20934 | 2 | 3 | 0.22 | K.TAWTFSR.K | Lactoylglutathione lyase | glo1 |
| P02747 | 45 | 25941 | 3 | 3 | 0.17 | K.FQSVFTVTR.Q | Complement C1q subcomponent subunit C | c1qc |
| A6QQV6 | 45 | 79661 | 2 | 1 | 0.05 | R.VIIPPLELER.C | Protein arginine N-methyltransferase 7 | prmt7 |
| Q8R3H9 | 44 | 44679 | 2 | 1 | 0.10 | K.LKPGHLK.A | Tetratricopeptide repeat protein 4 | tto4 |
| Q68DL7 | 43 | 77871 | 4 | 1 | 0.06 | R.LPAPELK.E | Uncharacterized protein C18orf63 | c18orf63 |
| Q4R5E9 | 43 | 70874 | 1 | 1 | 0.06 | R.IILEALR.Q | Secretogranin-2 | scg2 |
| A4Z6H0 | 43 | 50343 | 2 | 3 | 0.09 | R.VTVVLGAQWGDEGK.G | Adenylosuccinate synthetase isozyme 1 | adssl1 |
| Q5R8Y6 | 42 | 76640 | 3 | 1 | 0.06 | R.IEPSPYK.F | Transmembrane 9 superfamily member 2 | tm9sf2 |
| Q6P6V1 | 41 | 69685 | 3 | 1 | 0.06 | K.SSSELGMIFNER.D | Polypeptide N-acetylgalactosaminyltransferase 11 | galnt11 |
| Q6ZQ82 | 41 | 92610 | 2 | 1 | 0.05 | R.IVGVNSRVQK.L | Rho GTPase-activating protein 26 | arhgap26 |
| Q8R238 | 40 | 35078 | 1 | 4 | 0.13 | R.RLEGEGAEVQLTGK.V | Serine dehydratase-like | sdsl |
| Q2MHN2 | 40 | 21389 | 1 | 5 | 0.21 | K.ELGDHVTNLR.K | Ferritin heavy chain | fth1 |
| Q96QS6 | 39 | 43137 | 1 | 2 | 0.10 | K.TTKKPFAIK.V | Serine/threonine-protein kinase H2 | pskh2 |
| Q6W3E5 | 39 | 72363 | 1 | 1 | 0.06 | R.LVSIETLAK.N | Glycerophosphodiester phosphodiesterase domain-containing protein 4 | gdpd4 |
| Q9BXB4 | 37 | 84188 | 3 | 1 | 0.05 | K.ISLSNHYK.N | Oxysterol-binding protein-related protein 11 | osbpl11 |
| Q7TQD2 | 37 | 23698 | 1 | 5 | 0.19 | R.LTDTSKFTGSHK.E | Tubulin polymerization-promoting protein | tppp |
| Q4R362 | 37 | 11360 | 1 | 9 | 0.44 | R.ISGLIYEETR.G | Histone H4 | qtsa |
| O88879 | 37 | 142798 | 2 | 1 | 0.03 | K.KEDLPAEAHSIIK.E | Apoptotic protease-activating factor 1 | apaf1 |
| Q8NEM1 | 37 | 63275 | 1 | 1 | 0.07 | K.VIHTGEK.S | Zinc finger protein 680 | znf680 |
| P97324 | 37 | 59502 | 3 | 1 | 0.07 | -.MAEQVTLSR.T+ Oxidation (M) | Glucose-6-phosphate 1-dehydrogenase 2 | g6pd2 |
| Q0VGK2 | 37 | 59722 | 4 | 2 | 0.07 | K.IINLLGFPGDR.L | Tetratricopeptide repeat protein 39C | ttc39c |
| Q13410 | 37 | 59383 | 3 | 2 | 0.07 | R.GRATLVQDGIAK.G | Butyrophilin subfamily 1 member A1 | btn1a1 |
| Q2EN76 | 36 | 17257 | 1 | 11 | 0.27 | R.VMLGETNPADSKPGTIR.G+ Oxidation (M) | Nucleoside diphosphate kinase B | nme2 |
| Q9WVB0 | 36 | 21848 | 5 | 4 | 0.21 | -.MNGGGKAEK.E+ Oxidation (M) | RNA-binding protein with multiple splicing | rbpms |
| P48065 | 35 | 70380 | 1 | 1 | 0.06 | R.EGLIAGEK.E | Sodium- and chloride-dependent betaine transporter | slc6a12 |
| Q2M2U5 | 35 | 19844 | 1 | 3 | 0.23 | R.ERAVVK.L | IQ domain-containing protein F2 | iqcf2 |
| Q9BE72 | 35 | 67948 | 1 | 2 | 0.06 | R.ALSDATEELTVIK.S | Solute carrier family 2, facilitated glucose transporter member 12 | slc2a12 |
| Q3SWX5 | 35 | 88484 | 1 | 1 | 0.05 | K.DNTAGIFTRK.N | Cadherin-6 | cdh6 |
| Q8BHF7 | 34 | 62818 | 6 | 1 | 0.07 | K.FPSDLK.V | CDP-diacylglycerol-glycerol-3-phosphate 3-phosphatidyltransferase,  mitochondrial | pgs1 |
| Q4V8G5 | 34 | 41325 | 2 | 2 | 0.11 | R.SRIQQNLK.N | Septin-12 | sept12 |
| Q4R4W5 | 34 | 26669 | 1 | 3 | 0.17 | K.KAANGEIK.I | Isopentenyl-diphosphate Delta-isomerase 1 | idi1 |
| Q4FZT9 | 34 | 100768 | 1 | 1 | 0.04 | R.VGQAVDVVGQAGKPK.T | 26S proteasome non-ATPase regulatory subunit 2 | psmd2 |
| Q9NQX1 | 34 | 74699 | 2 | 1 | 0.06 | K.FPVKQALQR.H | PR domain zinc finger protein 5 | prdm5 |
| B7ZNG4 | 33 | 72650 | 2 | 1 | 0.06 | R.TLALRQR.L | Tastin | troap |
| Q9EP69 | 33 | 67361 | 3 | 1 | 0.06 | -.MAAAAYEHLK.L+ Oxidation (M) | Phosphatidylinositide phosphatase SAC1 | sacm1l |
| Q8WUM4 | 32 | 96469 | 1 | 1 | 0.04 | K.KDNDFIYHDR.V | Programmed cell death 6-interacting protein | pdcd6ip |
| Q3SWU0 | 32 | 39331 | 1 | 1 | 0.11 | R.ALQYLR.I | RISC-loading complex subunit TARBP2 | tarbp2 |
| Q8TD55 | 32 | 53593 | 2 | 1 | 0.08 | K.ILSEKLK.A | Pleckstrin homology domain-containing family O member 2 | plekho2 |
| A3KN27 | 32 | 60420 | 2 | 2 | 0.07 | K.GGFSGHSAVVLR.K | Keratin, type II cytoskeletal 74 | krt74 |
| P48506 | 32 | 73363 | 2 | 1 | 0.06 | K.HPRFSTLTR.N | Glutamate-cysteine ligase catalytic subunit | gclc |
| B2RZ78 | 32 | 20594 | 1 | 5 | 0.22 | K.TLAGDVHIVR.G | Vacuolar protein sorting-associated protein 29 | vps29 |
| Q3T140 | 31 | 10983 | 1 | 16 | 0.45 | K.GVQGIIVVNTEGIPIK.S | Dynein light chain roadblock-type 1 | dynlrb1 |
| Q8K1R7 | 31 | 108501 | 4 | 1 | 0.04 | R.GAFGEATLYR.R | Serine/threonine protein kinase Nek9 | nek9 |
| Q5NVP9 | 31 | 37259 | 3 | 3 | 0.12 | K.TSGLQQKNVDVK.T | Mortality factor 4-like protein 1 | morf4l1 |
| Q9JI24 | 30 | 21174 | 1 | 5 | 0.22 | K.QLDIEVALAK.A | Interleukin-24 | il24 |
| O35774 | 30 | 94630 | 2 | 2 | 0.05 | K.GAAGPRSDGELNLENLEEK.E | A-kinase anchor protein 4 | akap4 |
| Q9H7P6 | 30 | 35919 | 1 | 3 | 0.12 | R.GTDQSTMPEVK.D | Multivesicular body subunit 12B | mvb12b |
| Q8CG76 | 30 | 40954 | 1 | 2 | 0.11 | R.AVGRAAVR.S | Aflatoxin B1 aldehyde reductase member 2 | akr7a2 |
| A2VCK2 | 30 | 37963 | 2 | 2 | 0.12 | K.LLTEKVK.L | Doublecortin domain-containing protein 2B | dcdc2b |
| A4FUB0 | 30 | 73548 | 1 | 1 | 0.06 | K.ENELYHQIMK.S | Uncharacterized protein C5orf34 homolog | c5orf34 |
| Q9CXW2 | 30 | 41258 | 2 | 3 | 0.11 | -.MAAVRTPLSLWR.F | 28S ribosomal protein S22, mitochondrial | mrps22 |
| Q6IRU5 | 29 | 25248 | 1 | 3 | 0.18 | K.VTEQEWR.E | Clathrin light chain B | cltb |
| Q8NGL2 | 29 | 35272 | 1 | 2 | 0.13 | K.MGSAEGR.H+ Oxidation (M) | Olfactory receptor 5L1 | or5l1 |
| Q4KLM6 | 29 | 80472 | 1 | 1 | 0.05 | R.HKLEAELIK.S | Prolyl 3-hydroxylase 2 | p3h2 |
| Q9WUH5 | 29 | 56607 | 2 | 2 | 0.08 | R.ETGQHGAHTVR.F | Tripartite motif-containing protein 10 | trim10 |
| O60336 | 29 | 165188 | 1 | 1 | 0.03 | R.HEASLQAPSPGALLSR.E | Mitogen-activated protein kinase-binding protein 1 | mapkbp1 |
| Q6IUU3 | 29 | 83004 | 3 | 1 | 0.05 | K.DINEFFTR.S | Sulfhydryl oxidase 1 | qsox1 |
| Q28960 | 29 | 31949 | 1 | 2 | 0.14 | K.TNFMGTR.N+ Oxidation (M) | Carbonyl reductase [NADPH] 1 | cbr1 |
| Q9BZB8 | 29 | 63245 | 1 | 2 | 0.07 | R.INAILDNSLDFSR.V | Cytoplasmic polyadenylation element-binding protein 1 | cpeb1 |
| Q6P9L6 | 28 | 160895 | 2 | 1 | 0.03 | K.QQQEHVTQLSDLEK.Q | Kinesin-like protein KIF15 | kif15 |
| Q53GT1 | 28 | 72449 | 1 | 2 | 0.06 | R.SLLLEPPRGTPDR.S | Kelch-like protein 22 | klhl22 |
| Q5RER6 | 26 | 38650 | 1 | 3 | 0.11 | R.CHAPENVELALR.E | Serine/threonine-protein kinase PDIK1L | pdik1l |
