# Supplementary material for: Proteins from toad’s parotoid macroglands: do they play a role in gland functioning and chemical defence?
Source: Front Zool. 2023 Jun 16;20:21. doi: 10.1186/s12983-023-00499-8 (PMC10273630; doi:10.1186/s12983-023-00499-8)
Supplement: Supplementary file 2 — Additional file 2. Figures A1-A5. Fig. A1 STRING protein-protein interaction network predicted for proteins identified in the extract from parotoids of the common toad Bufo bufo based on homology to Xenopus tropicalis. Red balls represent proteins involved in metabolic processes, while lines show interactions between proteins. The thicker the line, the stronger the interaction. Abbreviations within the balls represent protein labels. Full protein names and their labels are provided in Table A1 in Additional file 1. Fig. A2 Number of proteins involved in metabolic processes predicted based on the Gene ontology enrichment analysis of proteins identified in the extract from parotoids of the common toad Bufo bufo. Fig. A3 Molecular functions and number of proteins predicted based on the Gene ontology enrichment analysis of proteins identified in the extract from parotoids of the common toad Bufo bufo. Fig. A4 Number of proteins involved in signalling pathways predicted based on the KEGG analysis of proteins identified in the extract from parotoids of the common toad Bufo bufo. Fig. A5 Localisation of proteins identified in the extract from parotoids of the common toad Bufo bufo within the cell based on the Gene ontology component analysis [file 12983_2023_499_MOESM2_ESM.docx]

**Proteins from toad’s parotoid macroglands: Do they play a role in gland functioning and chemical defence?**

Krzysztof Kowalski^1*^, Paweł Marciniak^2^ and Leszek Rychlik^3^

^1^Department of Vertebrate Zoology and Ecology, Institute of Biology, Faculty of Biological and Veterinary Sciences, Nicolaus Copernicus University, Lwowska 1, Toruń, 87-100, Poland, e-mail: k.kowalski@umk.pl,: tel. +48 56 611 4910

^2^Department of Animal Physiology and Developmental Biology, Institute of Experimental Biology, Faculty of Biology, Adam Mickiewicz University, Uniwersytetu Poznańskiego 6, Poznań, 61-614, Poland, e-mail: pmarcin@amu.edu.pl, tel.: +48 61 829 5926

^3^Department of Systematic Zoology, Institute of Environmental Biology, Faculty of Biology, Adam Mickiewicz University, Uniwersytetu Poznańskiego 6, Poznań, 61-614, Poland, e-mail: leszek.rychlik@amu.edu.pl, tel.: +48 61 829 5751

*Correspondence: k.kowalski@umk.pl (K.K.)

**Results**


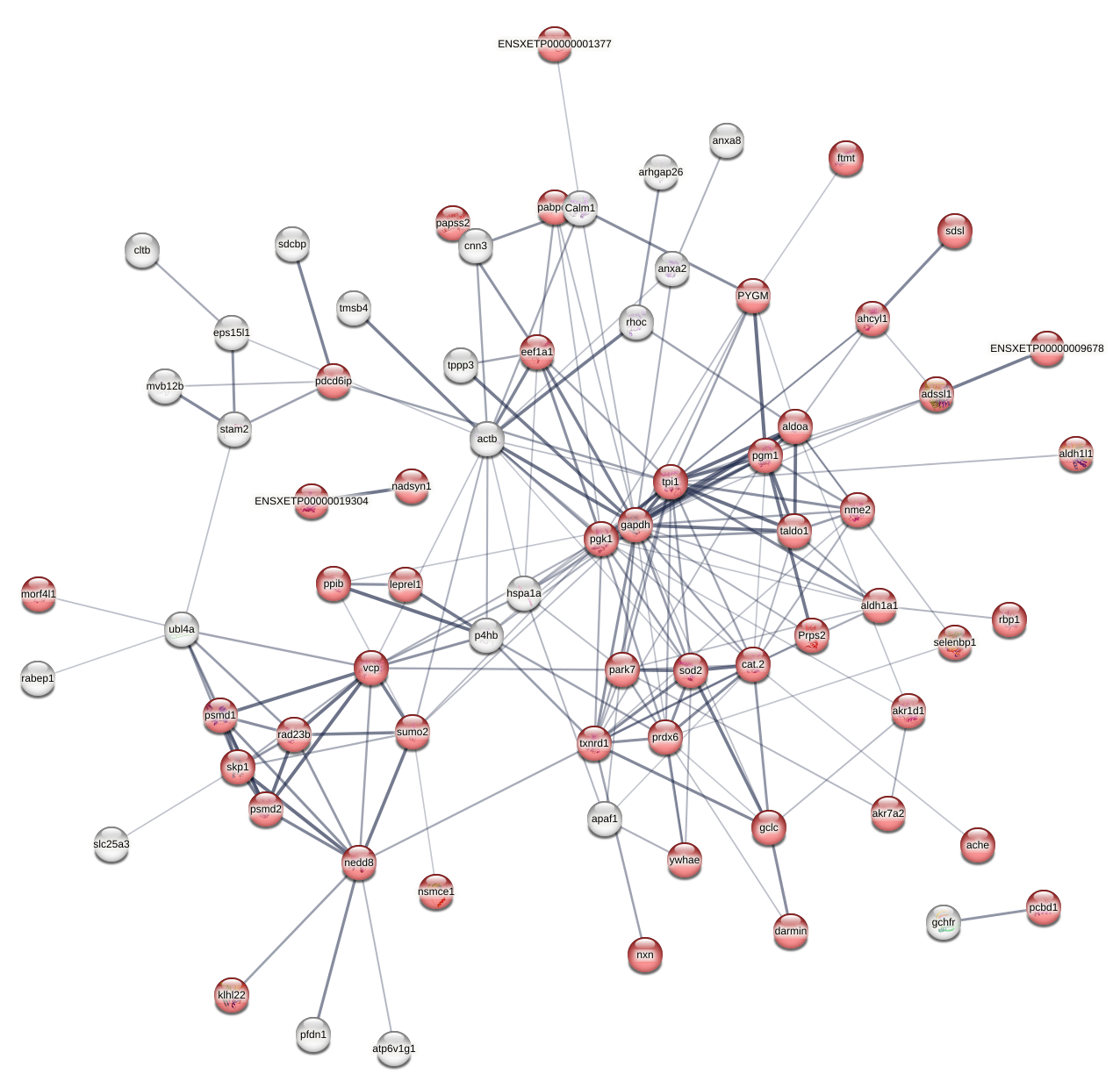


**Fig. A1** STRING protein-protein interaction network predicted for proteins identified in the extract from parotoids of the common toad *Bufo bufo* based on homology to *Xenopus tropicalis*. Red balls represent proteins involved in metabolic processes, while lines show interactions between proteins. The thicker the line, the stronger the interaction. Abbreviations within the balls represent protein labels. Full protein names and their labels are provided in Table A1 in Additional file 1.


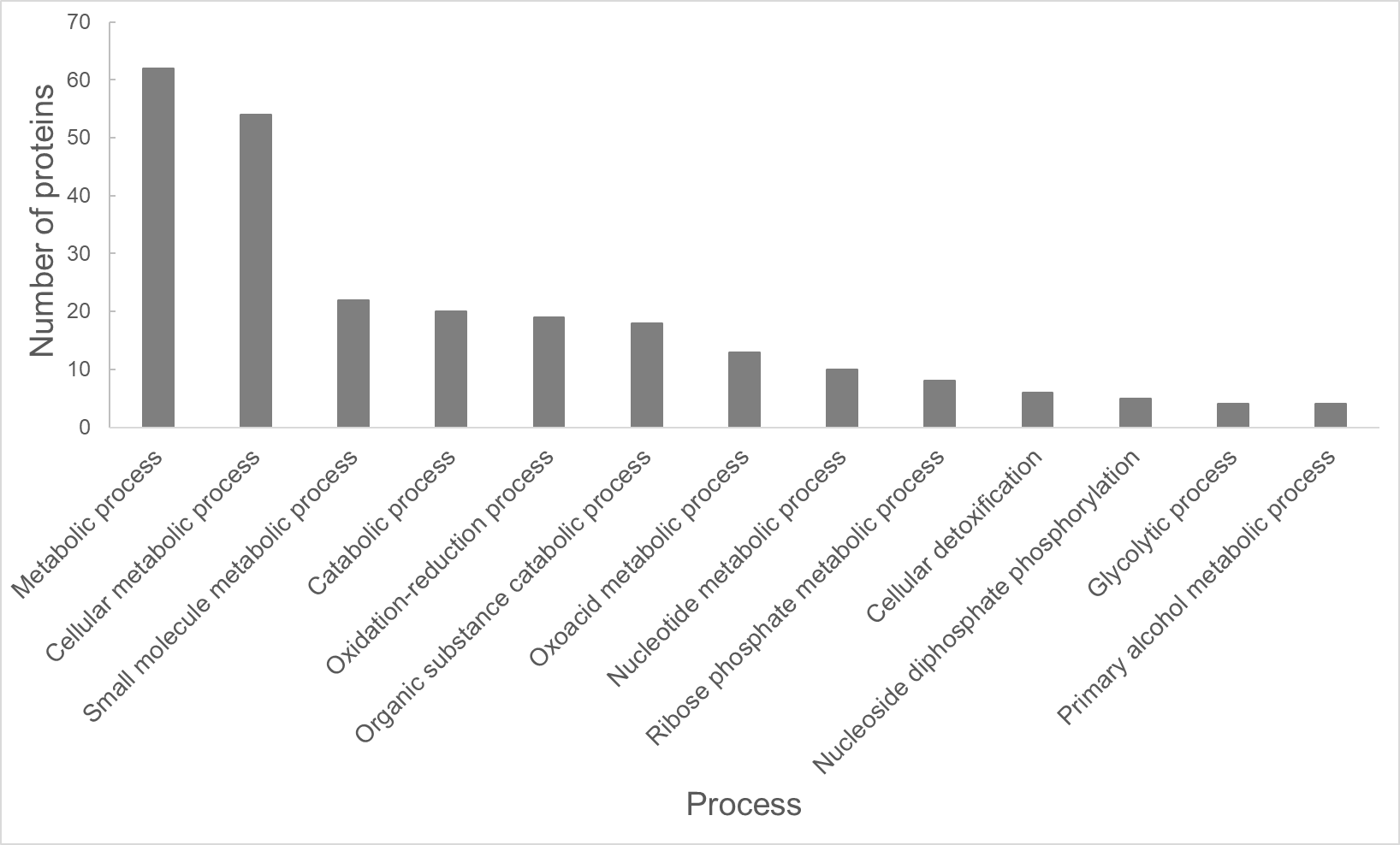


**Fig. A2** Number of proteins involved in metabolic processes predicted based on the Gene ontology enrichment analysis of proteins identified in the extract from parotoids of the common toad *Bufo bufo*


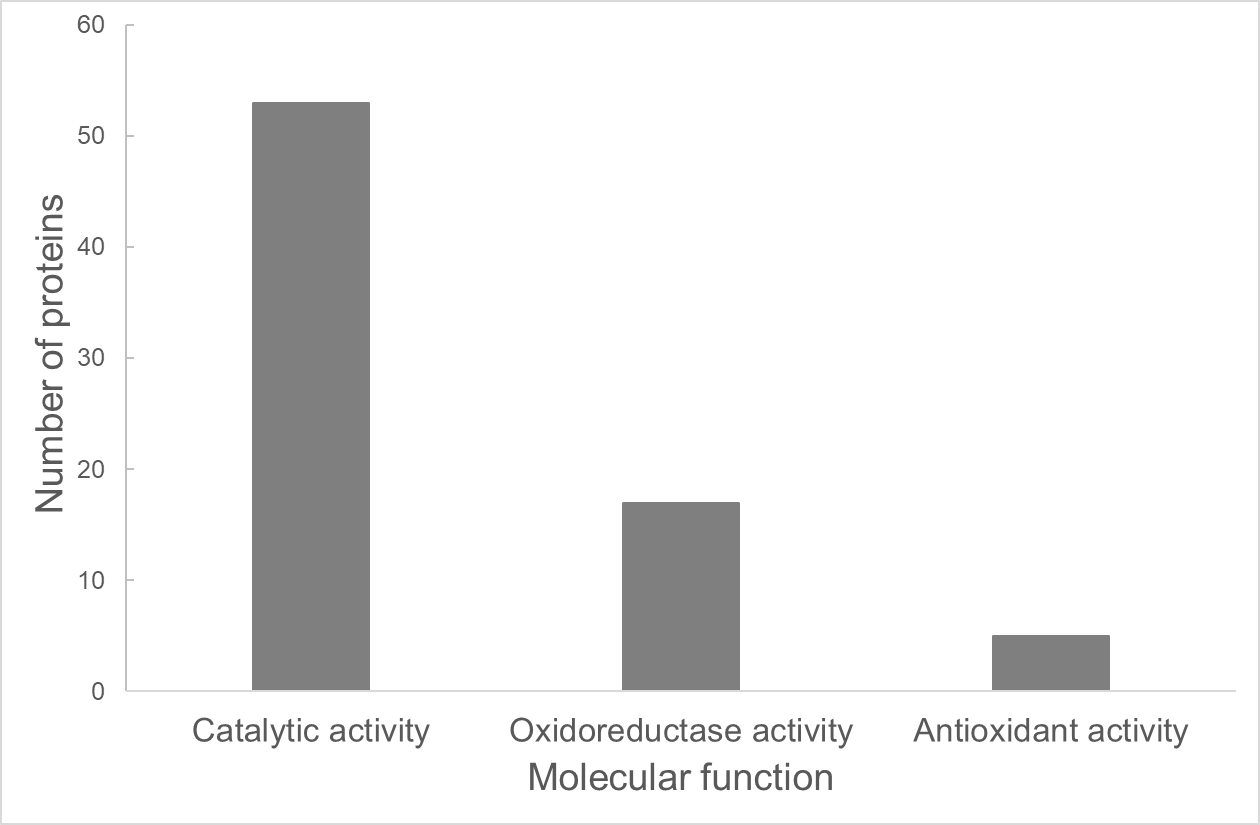


**Fig. A3** Molecular functions and number of proteins predicted based on the Gene ontology enrichment analysis of proteins identified in the extract from parotoids of the common toad *Bufo bufo*


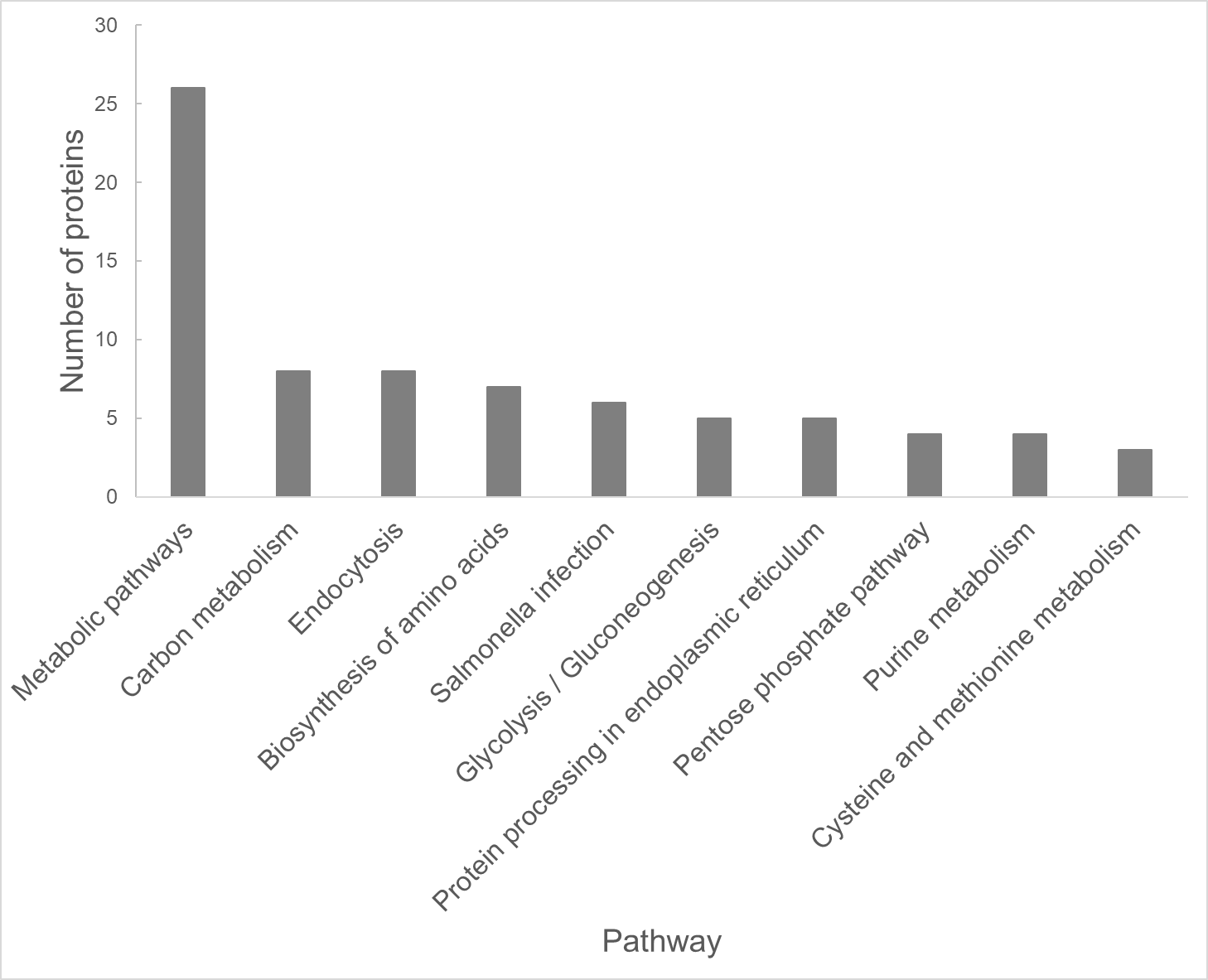


**Fig. A4** Number of proteins involved in signalling pathways predicted based on the KEGG analysis of proteins identified in the extract from parotoids of the common toad *Bufo bufo*

**
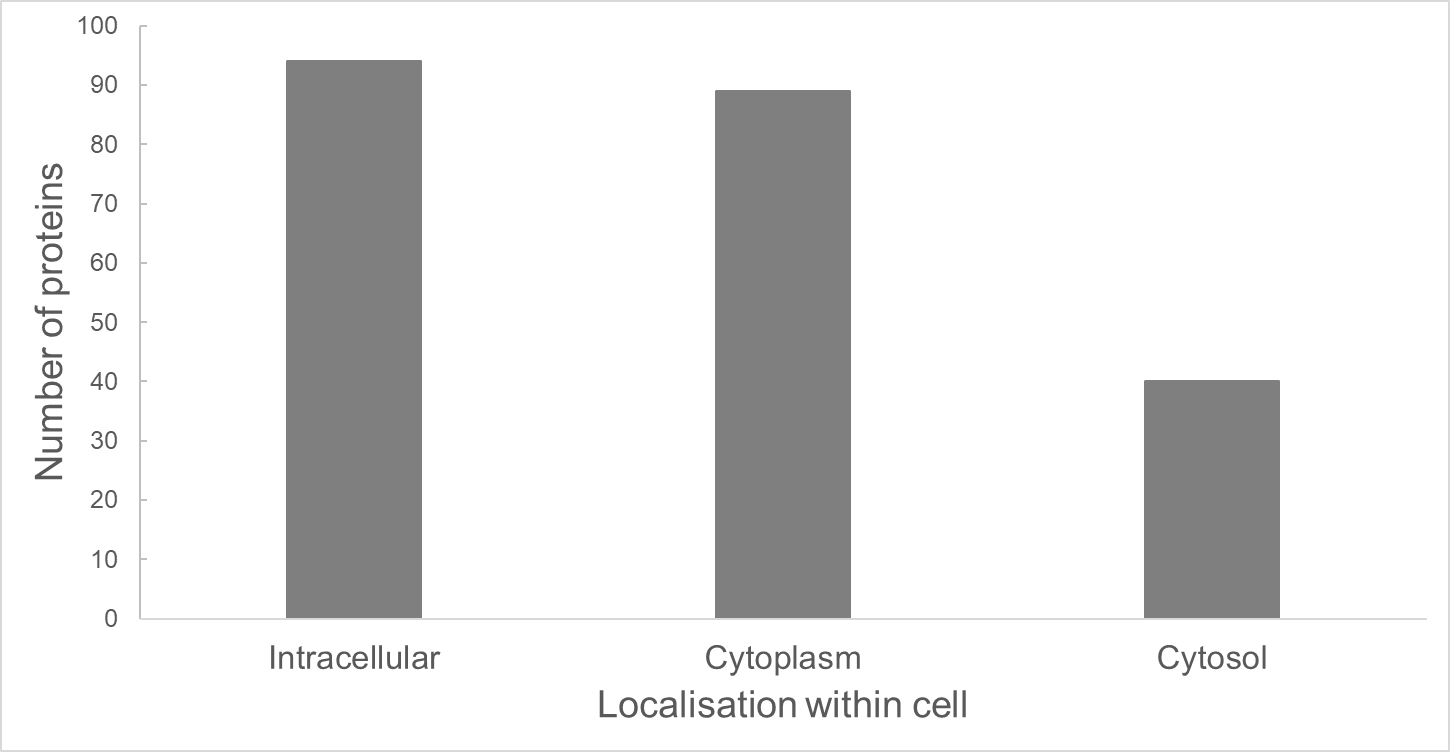
**

**Fig. A5** Localisation of proteins identified in the extract from parotoids of the common toad *Bufo bufo* within the cell based on the Gene ontology component analysis
